# De novo design of anticancer 4-thiazolidinone derivatives: a generative framework shaped by activity cliffs

**DOI:** 10.1186/s13321-026-01216-3

**Published:** 2026-05-11

**Authors:** Tomasz Szostek, Maciej Wiśniewski, Davide Ballabio, Viviana Consonni, Dariusz Plewczyński, Daniel Szulczyk

**Affiliations:** 1 Chair and Department of Biochemistry, Medical University of Warsaw, 02-097 Warsaw, Poland; 2 Laboratory of Bioinformatics and Computational Genomics, Faculty of Mathematics and Information Science, Warsaw University of Technology, Koszykowa 75, 00-662 Warsaw, Poland; 3 Milano Chemometrics and QSAR Research Group, Department of Earth and Environmental Sciences, University of Milano Bicocca, Piazza della Scienza 1, 20126 Milano, Italy; 4 Laboratory of Functional and Structural Genomics, Centre of New Technologies, University of Warsaw, Banacha 2C, 02-097 Warsaw, Poland

**Keywords:** Activity cliffs, Drug discovery, 4-thiazolidinones, QSAR, Machine learning, Anticancer

## Abstract

Activity cliffs (AC) correspond to large potency differences between highly similar compounds and pose a persistent challenge for both predictive modeling and de novo molecular design, particularly in small and underexplored areas of the chemical space. In this study, we introduce an AC-aware generative framework for the de novo design of anticancer thiazolidinone derivatives relevant to non-small cell lung cancer. We identify difficult regions of structure–activity landscape and extract chemical pattern signals hidden in its discontinuities directly from AC pairs using a dedicated algorithm. These signals are incorporated into a fragment-based generative pipeline to guide molecular construction and candidate selection. Compared with a standard QSAR-guided approach, the proposed framework yields a higher fraction of candidates with favorable docking profiles across multiple cancer-relevant targets and produces ligands with more stable binding modes in molecular dynamics simulations. Among the top five candidates selected by a molecular dynamics, multi-metric consensus combining docking affinity, binding stability (ligand and pocket RMSD/RMSF), and energetic criteria, four were generated using the AC-aware CAFE LATE strategy. The two highest-ranked hits exhibited binding energies of − 7.78 and − 7.70 kcal/mol, markedly more favorable than the native reference (− 4.09 kcal/mol), and consistently outperformed native ligands across all evaluated stability metrics. Those results suggest that explicit use of AC information can improve the quality of de novo generated small-molecule candidates in data-limited settings.

**Supplementary Information:**

The online version contains supplementary material available at 10.1186/s13321-026-01216-3.

## Scientific Contribution statement

This study introduces a new AC-informed small-molecule design framework that explicitly exposes crucial structure–activity information hidden in steep regions of the SAR landscape and uses it as an operationalized design signal. Our algorithm converts AC knowledge into reverse QSAR and generation leverage, enabling exploration of unknown areas of the chemical space, and discovery of the best performing candidates supported in molecular dynamics simulations. To the best of our knowledge, this is the first reverse QSAR and fragment-based generative framework to operationalize AC information at the fragment level within a unified machine learning workflow.

**Supplementary Information:**

The online version contains supplementary material available at 10.1186/s13321-026-01216-3.

## Introduction

Lung cancer remains the most diagnosed cancer and the leading cause of cancer death worldwide. In 2022 it accounted for ~ 2.48 million new cases (12.4% of all cancers) and ~ 1.82 million deaths (18.7%) [1]. Despite advances in screening and therapeutics, the absolute global burden continues to rise, driven by population aging and growth alongside persistent exposures to major risk factors. The World Health Organization projects more than 35 million new cancer cases per year by 2050 [2]. Consequently, the clinical management of the predominant histologic category of non-small cell lung cancer (NSCLC) centers on comprehensive biomarker testing to guide targeted and individualized therapies and immunotherapies, as reflected in contemporary international guidelines [3]. Nevertheless, durable disease control in advanced NSCLC remains uncommon because relapse and progression are frequent, reflecting tumor heterogeneity and acquired resistance (e.g. secondary epidermal growth factor receptor mutation [4]), bypass signaling [5], and lineage transformation [6]. Recent randomized trials have moved targeted and immune therapies into earlier stages, for example perioperative durvalumab improved event-free survival and pathologic complete response [7], and adjuvant alectinib prolonged disease-free survival after resection of anaplastic lymphoma kinase positive disease (ALINA) [8], but they have not eliminated relapse. Given the clinical reality and therapeutic pressure, there is a profound need for new small-molecules discovery, which could expand possibilities of NSCLC selective treatment.

Thiazolidinone is a widely investigated, pharmacologically versatile scaffold and remains a promising core chemotype for anticancer lead discovery. As summarized in prior reviews, thiazolidinones have attracted sustained attention from medicinal chemists owing to their broad spectrum of biological activities, including anticancer effects, and their synthetically accessible substitution patterns [9, 10]. Recent studies further underscore the anticancer potential of this pharmacophore. Podolak et al. (2025) reported a new series of highly active derivatives with lead achieving IC_50_ = 0.85 µM in MCF-7 human breast adenocarcinoma cells and 0.28 µM in Jurkat human T-cell leukemia cells, while exhibiting markedly weaker effects in normal human dermal fibroblasts (HDF IC_50_ = 16.9 µM), indicating useful selectivity [11]. In a complementary series, Shawky et al. (2023) disclosed 2,3-diaryl-1,3-thiazolidin-4-ones with IC_50_ values ranging from 0.02 to 17.02 µM across MCF-7 human breast adenocarcinoma cells, HT-29 human colorectal adenocarcinoma cells, and A2780 human ovarian carcinoma cells, along with in vivo tumor growth suppression in an Ehrlich solid carcinoma model [12]. Most importantly, several studies show that thiazolidinone derivatives exhibit measurable cytotoxicity against NSCLC models. For example, novel 4-thiazolidinone-based compounds were screened for in vitro anticancer activity and demonstrated inhibitory effects on the A-549 human cell line in MTT assays, with IC_50_ values in the low-to-mid micromolar range [13]. Moreover, related thiazolidinone hybrids have shown apoptosis induction and cell cycle arrest in A-549 cells, reinforcing the relevance of this scaffold to NSCLC drug discovery [14].

Yet the thiazolidinone chemical space understood as the conceptual space of compounds with common chemical motif, remains underexplored, particularly along substituent vectors relevant to potency, selectivity, and drug-like properties. This motivates a systematic, structure-guided expansion in search of the new effective anticancer agents.

Modern in silico methods, including Artificial Intelligence (AI) powered frameworks, have become a standard tool in medicinal chemistry for de novo small-molecules generation, giving a promise of effective digital data processing and analysis. These approaches typically rely on predictive models trained on large chemical databases, which learn structure–property and structure–activity relationships and subsequently generate or prioritize new molecular structures with desired biological profiles [15]. Statistically, discovery phase of active molecule lasts from 3 to 6 years, but applying AI driven computational aided drug design (CADD) can accelerate the process by even 2 years. Moreover, it can shorten the clinical trial duration by ~ 15—30% and significantly rise drug candidate’s early-stage success (Phase I 80–90% vs historical 40–65%) [16, 17], as evidenced by small-molecule TNIK inhibitor INS018_055 which was designed using AI powered methodology and has successfully reached 2a phase of clinical trial targeting fibrosis [18]. Despite undeniable progress, several assessments caution that the real-world impact of AI on drug discovery remains uneven indicating necessary improvements in data quality management and models architecture [19].

From computational chemistry perspective, building AI models that capture a continuous structure–activity relationship (SAR) and reliable performance is primarily disrupted by pairs of very similar compounds with large potency differences ($\Delta p$)- Activity Cliffs (AC) [20, 21]. These discontinuities function as outliers that break the apparent correlation between shared chemical motifs and biological effects, where even single-atom edits can yield ≥ tenfold potency shifts [22]. As a result, generative and predictive models with tendency to generalization often produce overly optimistic estimates and inflated accuracy metrics, wasting time and resources, and risking overlooking of relevant candidates [23]. Importantly, medicinal chemists view AC as a dense, high-gradient, information rich regions of SAR landscape, which can be operationalized in AI pipelines using dedicated techniques enhancing the models’ performance [24]. At the molecular representation level, methods such as ACtriplet [25], ACANT [26], MaskMol [27] and SiamACLoss [28] inject pairwise/contrastive AC information during model training, explicitly separating AC neighbors in latent space and improving sample efficiency and reliability. For evaluation, MoleculeACE formalized AC-aware benchmarking by introducing RMSE_cliff_ (Root Mean Square Error calculated on AC pairs) and subset metrics [23], while ACNet [29] complements this by casting AC detection as a pairwise classification problem. Finally, de novo generative modeling was reshaped to explore difficult SAR region with Activity Cliffs Aware Reinforcement Learning (ACARL) [30] via contrastive objectives.

Quantitative Structure Activity Relationship (QSAR) models learn a statistical relationship between molecular structure and biological activity [31]. Once trained, they can predict unknown activity for new compounds within a defined Applicability Domain (AD), understood as a pre-specified region of chemical space where the model’s assumptions and uncertainty bounds justify using the prediction. Reverse QSAR (r-QSAR, often called inverse-QSAR) turns the problem around. Starting from desired property values or activity profiles, it searches the chemical space for structures likely to satisfy those targets, typically by coupling prediction model (QSAR) with a structure generator/decoder or chemically constrained mutation engine [32].

Rapid progress of AI and its implementation to QSAR modeling extended representation learning, and brought a great improvement to data interpretability, accelerating hypothesis testing in drug discovery pipelines. At the same time, extensive comparative studies in small-molecule frameworks, comparing Machine Learning (ML) and Deep Learning (DL) methods, consistently reveal superiority of simpler ML approaches. Rigorous evaluation under AC-aware conditions related to strict assessment metrics such as RMSE_cliff_ further highlights this advantage [23]. As evidenced in recent analysis conducted on 62,820 trained models, classical molecular representation aligned with ML methods can be the best choice, especially for smaller datasets [33].

In this study, we continue to expand thiazolidinones chemical space to identify new candidates against NSCLC [34]. To increase the likelihood of discovering highly active analogues in a limited and underexplored dataset, we introduce an AC-informed generative framework built around r-QSAR and fragment-based design. In contrast to previously reported AC-aware approaches, which primarily incorporate cliff information at the level of model training, latent-space shaping, loss design, or reinforcement-learning control, our strategy operates at the level of chemically interpretable building blocks. Specifically, it extracts directional SAR information from AC pairs, resolves this signal into fragment-level enrichment rules, and reinjects the corrected fragment knowledge into r-QSAR-guided molecular generation. To the best of our knowledge, this is the first study to combine r-QSAR, fragment-level AC knowledge extraction, and de novo design within one coherent small-molecule discovery workflow. This new approach turns AC from sources of model error into actionable signal, focusing search on high-value regions of chemical space and yielding a small library of thiazolidinone derivatives with higher probability of desirable potency gains against NSCLC.

## Methods

### Data acquisition and standardization

To establish a robust modeling foundation and define actionable chemical space for downstream analysis, we enforced strict data management rules. We retrieved all compounds, bearing the thiazolidinone scaffold with reported activity against A549 adenocarcinoma cell line, commonly used as a model of NSCLC from ChEMBL database (CHEMBL392), using the platform’s curated bioactivity records, and added 21 compounds previously synthesized by our team [34–36]. For endpoint consistency, we selected a single readout of IC_50_ values, derived exclusively from MTT colorimetric assay, and converted all measurements to pIC_50_, in common nM unit for concentration [38]:

1 $$p{IC}_{50}=-{log}_{10}({IC}_{50}\left[M\right])=9-{log}_{10}({IC}_{50}\left[nM\right])$$

This choice aligns with best practice for harmonizing potency scales and leverages ChEMBL’s conventions for comparability across studies. The dataset encoded as canonical SMILES was subjected to a standard curation workflow to minimize artifacts and leakage: removal of exact duplicates, resolution of tautomeric variants, stripping salts/solvates, and exclusion of structurally implausible or ill-specified records (“odd compounds”), though molecular weight (MolWt < 900 Da) and LogP < 8 filters. This follows established recommendations that rigorous, transparent curation is a precondition for reliable QSAR/ML modeling [39]. For subsequent classification tasks, each chemical of the cleaned set was binarized to an activity label ${y}_{i}$ using the common cheminformatics threshold ${IC}_{50}$ ≤10 µM (= active, otherwise inactive), a cutoff frequently adopted in benchmarking and target-agnostic screens [40]:

2 $${y}_{i}=\left\{\begin{array}{c}1\\ 0\end{array} \begin{array}{c}if {IC}_{{50}_{i}}\le 10 000 nM\\ else\end{array}\right.$$

### Chemical space and structure activity landscape

To align with community standards, we define AC as a pair of compounds with high 2D structural similarity computed on Morgan fingerprints ECFP4 (radius = 2, length = 2048) and Jaccard Tanimoto similarity index $\uptau \ge 0.8$, combined with a $\Delta p{IC}_{50}\ge$ 1.0 (10- fold difference in IC_50_) using RDKit [23, 41, 42]. For a dataset of *n* valid molecules, all unordered pairs ($i,j$) with $i <j$ are enumerated. For each pair, we compute Tanimoto similarity ${s}_{ij}$ and potency difference ΔpIC_50_. A pair is retained as an AC if it meets both thresholds: high 2D similarity (${s}_{ij}$ ≥ τ, default τ = 0.80) and large potency contrast ($\Delta p{IC}_{50}\ge$ δ, default δ = 1.0 on ${IC}_{50}$). For every retained pair we quantify AC severity with Structure Activity Landscape Index $SALI$ defined as:

3 $${SALI}_{ij}= \frac{\Delta p{IC}_{50}}{1-{s}_{ij} +\varepsilon }$$

where ${s}_{ij}$ is Tanimoto similarity and ε = 1 × 10⁻⁶ is added in the denominator to guard against division by zero for near-identical pairs. $SALI$ is used strictly as a diagnostic in this step and later as a weight in fragment enrichment in r-QSAR.

Two complementary visual diagnostics are produced directly from the fingerprints and potency values (these maps are only chemical space visualizations and are not used by any predictive model):

- Structure Activity Similarity (SAS) map: $SALI$ scatter plots $\Delta p{IC}_{50}$ versus Tanimoto for all pairs, using a continuous colormap for $SALI$ values on the subset that meets AC criteria. The similarity and potency thresholds (τ, δ) are drawn as dashed reference lines to delimit the AC region [43].
- t-SNE embedding with AC overlay: projects molecule level fingerprints to two dimensions using t-SNE [44]. Points are colored by $p{IC}_{50}$, and molecules participating in at least one AC (present in mol_i_ or mol_j_) are overlaid as highlighted markers.
- Multidimensional Scaling (MDS**):** projects molecular fingerprints into two dimensions using classical metric multidimensional scaling based on pairwise dissimilarities derived from ECFP4 fingerprints. The dissimilarity matrix is computed as 1—Tanimoto similarity, and molecules are embedded such that inter-point distances best preserve the original dissimilarities in the low-dimensional space. Points are colored by $p{IC}_{50}$ values, while molecules participating in at least one AC pair are marked as triangles. This representation provides a global, distance-preserving baseline view of the chemical space, complementary to the nonlinear t-SNE projection [45].

### Machine learning models

Both the r-QSAR and prediction-QSAR model were trained on 9 classical ML engines using scikit learn and imbalanced libraries [45]: logistic regression (LogReg), linear support vector machine (Linear SVM), radial basis function support vector machine (RBF-SVM; SVC), k-nearest neighbors (k-NN), random forest (RF), balanced random forest (BRF), ExtraTrees, gradient boosting (GB), XGBoost, and CatBoost.

### Reverse QSAR

We trained a r-QSAR classifier across nine distinct ML engines to identify the best one suited for the task: mining chemical patterns mostly accountable for anticancer activity against A549 cancer cell line. All compounds in the final thiazolidinone dataset were defragmented to chemically meaningful fragments using RDKit BRICS [42]. Those fragments were normalized to customized SMILES in which broken bonds are marked with attachment points [*]. We then build a binary fragment incidence matrix:

4 $$X\in {\left\{\mathrm{0,1}\right\}}^{N\times F}, {X}_{i, f}=1, if f is present in molecule i$$

where N is the number of molecules in training set, and $\mathcal{L}=\{{f}_{1},.,{f}_{F}\}$ is the vocabulary of normalized fragments (each f corresponds to one column of *X*). This representation is analogous to MACCS keys [47], and enables the classifier to learn SAR as fragment presence/absence and to rank fragments by learned importance [48]. This preserves the highest learned importance per normalized fragment. As a sanity filter, any fragment observed only in the inactive class has its importance set to 0 before selection to prevent one-class artifacts.

To avoid indirect data leakage from splitting AC pairs across folds, we generated ten independent repeats of a stratified, group-aware train/test split. For each repeat:

- We constructed the set of molecules that participate in at least one AC pair (from the $SALI$ analysis table).
- All molecules in this set were forced into train (AC pairs and their neighbors remain together, preserving the AC logic).
- The remaining non-AC molecules were split by class-stratified sampling to form test at the target proportion (default $20\%$) the rest joined train.
- Class labels remain balanced across repeats by stratification. Random seeds were varied per repeat to ensure independence.

This policy keeps AC relationships intact and prevents the model from “seeing one side of an AC” in train and the other in test set, which would inflate AC related performance. The operating point (classification threshold) used for thresholded metrics within each repeat was chosen by scanning probability scores $\rho \in [0; 1]$ and selecting a single operating threshold that maximized the designated objective (F1-score by default) on that repeat’s held-out set.

We summarize r-QSAR performance by the mean over the 10 repeats of the following metrics: AUPRC_active_ emphasizing performance on the positive class under imbalance (chosen as a primary metric), AUROC, Accuracy, Precision, Recall, F1- score, Matthews Correlation Coefficient (MCC). For each engine we report the mean of these metrics across the 10 repeats the best model is selected by maximizing mean AUPRC_active_. Tie-breakers, when needed, follow (in order) higher AUROC, higher MCC, and higher F1 at the chosen operating point.

The best r-QSAR model builds the fragment library from the ranked fragment-importance profile. Let $I(j)$ denote the learned importance of fragment $f$, and let $I(j)$ denote the corresponding importance value after sorting in descending order. The cumulative importance curve is then defined as:

5 $$g(k)= \frac{\sum_{j=1}^{k}{I}_{(j)}}{\sum_{j=1}^{F}{I}_{(j)}}, k=1,..,F$$

where $k$ is the number of top-ranked fragments included in the cumulative sum. Among all importance mass, $g(k)$ shows what fraction is already covered by the top-k fragments. The final number of important fragments is chosen by Kneedle detector [49], which searches for natural bend in importance curve (“the elbow”), and keep the compact, high-signal features.

This split strategy minimizes pairwise leakage but also renders the AC-free, and therefore easier test set, so the reported global metrics are overoptimistic. The performance is expected to degrade on real-world scenarios. To recover SAR signal hidden inside AC and correct r-QSAR fragments choices, we introduce a new logic Cliff Active Fragment Extraction (CAFE): a lightweight, training-only postprocessor. CAFE requires exactly three artifacts:

- A binary fragment incidence matrix $X$ from Eq. (4)
- SMILES list of the same N molecules, to map AC pairs to row indices in $X$.
- Table of listed AC pairs ($i,j$) with $\Delta p{IC}_{50}$ values, Tanimoto similarity ${s}_{ij}$, potency gap $\Delta {pIC}_{50}$, and $SALI$.

CAFE processes each AC pair in turn. It first checks that both molecules are present in the *X* and that $\Delta {pIC}_{50}$, values are available. The partner with the highest $\Delta {pIC}_{50}$ is treated as the more active molecule (${i}^{+}$), the partner with lower $\Delta p{IC}_{50}$ as inactive (${i}^{-}$), and the pair is oriented:

6 $${i}^{+}=argmax\{{pIC}_{50}\}, {i}^{-}=argmin\{{pIC}_{50}\}$$

Each molecule is represented as a binary vector over the same fragment set, where 1 means the fragment is present and 0 means it is absent. Let ${x}_{{i}^{+}}$ and ${x}_{{i}^{-}}$ denote the binary fragment-incidence vectors of the more active and less active analogues, respectively, corresponding to their rows in matrix $X$. CAFE performs element-wise subtraction to obtain a signed delta vector:

7 $$d_{i} j = x_{{(i^{ + } )}} - x_{{(i^{ - } )}} \left\{ { - 1,0, + 1} \right\}^{F}$$

which encodes fragment by fragment, whether a feature helps explain the potency flip for this pair. Each entry of ${d}_{ij}$ takes one of three values {−1, 0, + 1}. Positive one means the fragment is present only in the more active partner. Negative one means the fragment is present only in the less active partner. Zero means the fragment is present in both partners or in neither partner, nor carries information about the potency flip:

8 $${d}_{ij,}(f) = \left\{\begin{array}{c}+1,\\ 0,\\ -1,\end{array}\begin{array}{c} if { x}_{{i}^{+}(f)}=1 and {x}_{{i}^{-}(f)}=0 \\ if { x}_{{i}^{+}(f)}= {x}_{{i}^{-}(f)}\\ if {x}_{{i}^{+}\left(f\right)}=0 and {x}_{{i}^{-}(f)}=1\end{array}\right.$$

where *f* indexes the customized fragment vocabulary $\mathcal{L}={\{f}_{1},\dots , {f}_{F}\}$. Each $f$ corresponds to one column of the binary matrix $X$ (one feature). For each pair, a fragment that occurs only in the more active partner receives a positive contribution equal to the $SALI$ value of that pair. A fragment that occurs only in the less active partner receives a negative contribution of the same magnitude. Fragments with delta equal to zero are ignored. $SALI$ values are clipped to 99th percentile *q* to prevent single extreme pairs from dominating the sum:

9 $${\omega}_{ij}=min({SALI}_{ij}, c), c= {quantile}_{q}(\{{SALI}_{ij}:\left(i,j\right) \in Train\})$$

In the present dataset, this cap corresponded to a $SALI$ value of 14.92 and affected only 1 of 47 AC pairs (2.13%). Comparison of fragment rankings obtained with and without clipping showed complete preservation of the main enrichment signal (top 10 overlap = 10/10; top 20 overlap = 20/20; Spearman correlation = 1.000), indicating that clipping acted purely as a stabilization step preventing disproportionate influence from a rare extreme pair, rather than materially altering fragment prioritization.

The per-pair contribution is then:

10 $${e}_{ij}(f)={\omega}_{ij}{d}_{ij}(f)$$

This comparison is repeated across all valid AC pairs in the training data, and the contributions are accumulated per fragment. A fragment that is repeatedly unique to the more active side builds a large positive Enrichment Factor ${E}_{(f)}$) and becomes a candidate to add. A fragment that is repeatedly unique to the less active side builds a large negative score and becomes a candidate to remove:

11 $${E}_{(f)}=\sum_{(i,j)\in Train}{e}_{ij}(f)=\sum_{(i,j)\in Train}{\omega}_{ij}{d}_{ij}(f)$$

**Fig. 1:**
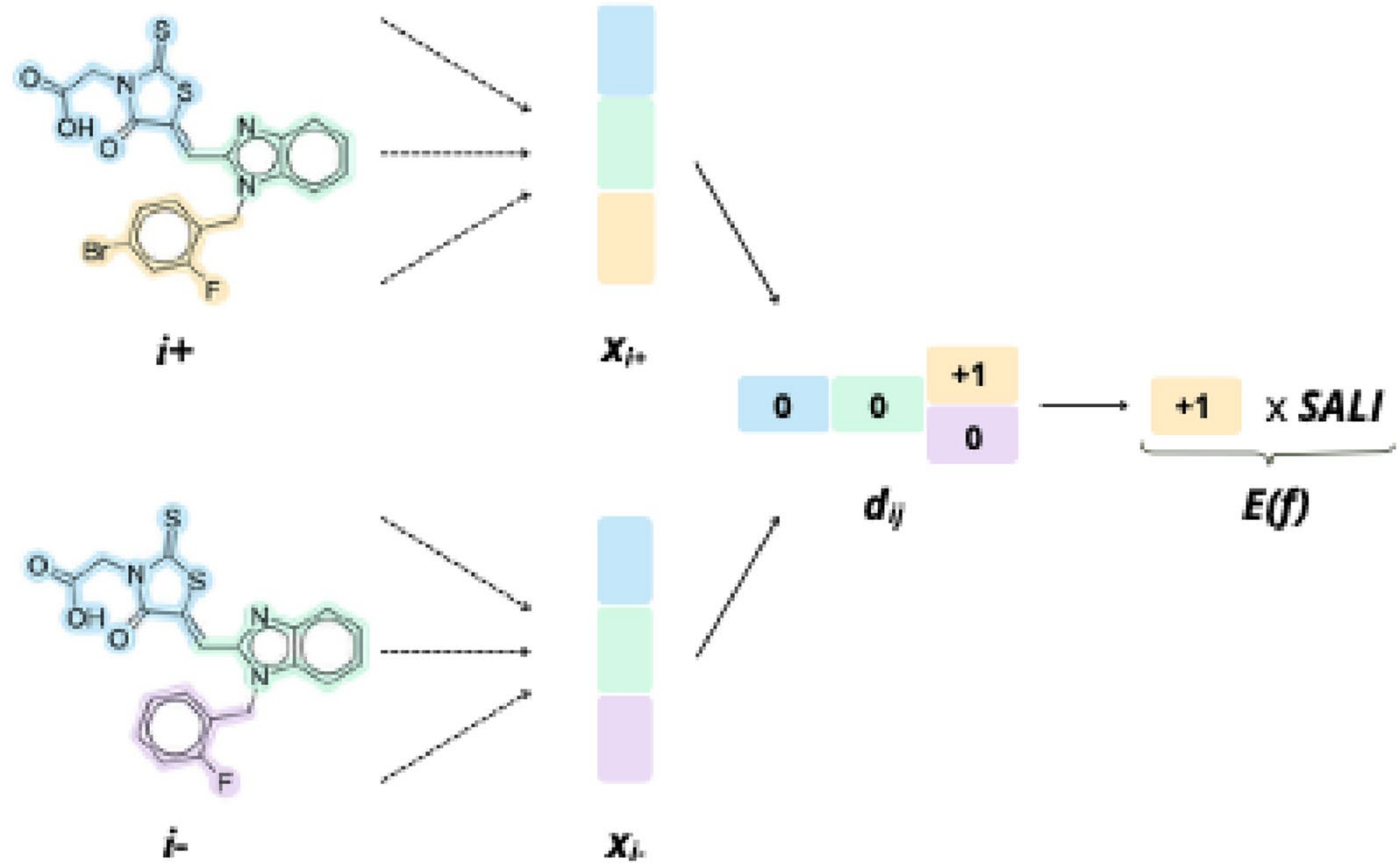
Operational principle of CAFE on a toy example. Fragment-level differences between the more and less potent analogues are encoded by the signed vector ${d}_{ij}={x}_{{i}^{+}} -{x}_{{i}^{-}}$, allowing AC pairs to generate positive, negative, or null fragment-enrichment contributions

The output is a structured table that maps each fragment to its total AC enrichment score and lists the supporting AC pairs with their row indices, so every recommendation has traceable provenance. Figure 1 illustrates the basic scoring on a toy example. Consider AC pair consisting of more potent analogue ${i}^{+}$ (active) and less potent analogue ${i}^{-}$ (inactive), represented by binary fragment presence vectors ${x}_{i+}$, and ${x}_{i-}$ respectively, over three fragments ${f}_{1}, {f}_{2} and {f}_{3}$ with a $SALI$ value of 3.2. Subtracting the binary vectors yields a directed ${d}_{ij}$ with entries -1, 0, or + 1. A value of + 1 indicates that the fragment is present only in the more active analogue and therefore contributes + 3.2 to its enrichment score. A value of -1 indicates that the fragment is present only in the less active analogue and therefore contributes -3.2. A value of 0 means that the fragment is shared by both analogues or absent from both and thus does not contribute to the potency flip. When such contributions are accumulated across many AC pairs, local SAR discontinuities are transformed into a stable fragment-level enrichment signal. The final fragment library is then corrected by CAFE through promotion of fragments repeatedly associated with the more active side of AC pairs and demotion of fragments repeatedly associated with the less active side.

### Prediction QSAR

We trained a binary classification QSAR across the same nine ML engines using four molecular representations: circular Morgan/ECFP fingerprints with radius 2 at 1024 and 2048 bits (hash-based, neighborhood-expansion), MACCS structural keys (classical 166-bit MDL keyset), and 31 physicochemical descriptors selected by Boruta feature selection algorithm from a pool of ≈1500 computed features (all-relevant wrapper over Random Forest) [50]. Continuous features are z-standardized, binary fingerprints are used as is. Performance is estimated over ten independent repeats. For each repeat, we build AC groups from the $SALI$ table and perform a stratified, group-aware split that keeps each AC component in the same fold (train or test), with test ≈ 20% and class proportions preserved [51]. Unlike r-QSAR (where test was AC free), here AC can appear in test, enabling AC-aware error estimates. After fitting on train, each model produces probabilities $\rho \in [\mathrm{0,1}]$. We select operating threshold $t*$ per repeat by grid search on a held-out validation split of train:

12 $$t*=argmax{MCC}_{(t)}$$

where ${MCC}_{(t)}$ denotes the Matthews Correlation Coefficient evaluated at decision threshold $t$ on the validation split.

Model selection prioritized AC-aware accuracy: we chose the configuration with the lowest and most stable ${RMSE}_{cliff}$ across repeats, the smallest mean and the narrowest 95% CI on AC compounds, as recommended by MoleculeACE benchmark [23]. Because our prediction task is binary (active vs inactive), we adapt this AC-aware evaluation principle and assess models on the AC subset using the squared error between predicted probabilities and binary labels- the Brier score [52], reported as its square root ($RMSE$). For each repeat $r$, let ${C}^{(r)}$ be the test molecules that belong to at least one AC pair, then:

13 $${RMSE}_{cliff}^{(r)}=\sqrt{\frac{1}{\left|{C}^{(r)}\right|}\sum_{i\in {C}^{(r)}}{({\widehat{p}}_{i}-{y}_{i})}^{2}}$$

This metric is a proper scoring rule for Bernoulli outcomes (minimized at the true class probability), hence it jointly rewards calibration and accuracy on the steep SAR region [53]. For aggregation across repeats, let ($r=1, \dots , R$), and ${c}_{(r)}= {RMSE}_{cliff}^{(r)}$, then:

14 $$\widetilde{c}=\frac{1}{R}{\sum}_{r=1}^{R}{c}_{(r)}$$

15 $$CI95\%=\left[{Q}_{2.5}, {Q}_{97.5}\right]$$

16 $$\mu = {Q}_{97.5}({c}_{(r)})-{Q}_{2.5}({c}_{(r)})$$

The best model is chosen based on the lowest final score (*Sc*) from aggregated mean ${RMSE}_{cliff}$ from ten repeats, combined with confidence interval width correction ($\mu$):

17 $$Sc= \widetilde{c} + \mu$$

This strategy allows to choose model with the lowest and most stable predictions on AC pairs.

### De novo* design*

To expand the thiazolidinone chemical space we use an island framework [54]. Each island is anchored on a distinct thiazolidinone core extracted from the final fragment library, and generation proceeds independently on each island, so coverage is spread across cores rather than collapsing onto a single neighborhood. Fragments and cores expose attachment points as [*] in SMILES. The generator aligns a library fragment to a compatible attachment on a core and builds a candidate molecule until no [*] attachment is left. Fragment sampling is two-phase. In the coverage phase, we iterate across the deduplicated fragment set on every core, so each core–fragment combination is tried at least once, fragment order is skewed to under-used pieces to prevent early collapse. After roughly 30% of the target is reached, we switch to a bandit phase: each fragment carries a probability that is updated from its recent post-generation performance. A small, fixed exploration mass is retained, so learning never shuts off diverse fragments. Island scheduling mirrors this logic. During coverage, islands are visited round-robin to ensure balanced growth. In bandit mode, island selection becomes performance-aware so that productive cores get proportionally more attempts without starving others.

Every candidate must pass basic medicinal chemistry gates before scoring. We require successful sanitization in RDKit terms (valence, aromaticity, formal charge consistency), full resolution of all placeholder atoms, a non-trivial size window (atom count 2–200), valid SMILES, and a molecular weight between 150 and 800 Da. Only molecules that satisfy these rules are forwarded to evaluation. After passing basic gates, candidate is transferred to multi-objective scoring consisting of the best selected QSAR classifier, QED [55] (Quantitative Estimate of Drug-Likeliness), RDKit implementation of Ertl–Schuffenhauer SA score (measuring synthetic accessibility), which was invert-normalized:

18 $$SA{\prime}=1-min(\frac{SA-1}{9}, 1)$$

where lower raw $SA$ scores indicate easier synthesis, and the transformed $SA{\prime}$ rescales this quantity so that higher values correspond to more favorable synthetic accessibility.

The aggregate score for molecule (m) used for ranking the best candidates is a convex combination:

19 $$\varphi \left(m\right)={\beta}_{QSAR}\widehat{p}\left(m\right)+ {\beta}_{SA}S{A}{\prime}\left(m\right)+{\beta}_{QED}QED\left(m\right)+ { \lambda }_{CAFE}B(m)$$

where $\widehat{p}(m)$ is the QSAR predicted probability, $B(m)$ is the CAFE LATE bonus defined below, and $\beta$ (${\beta}_{QSAR}= 0.4, {\beta}_{SA}= 0.4, {\beta}_{QED}=0.2$) are the tunable weights for each of the scorers. Then ${\lambda}_{CAFE}$ controls the contribution of the AC-aware CAFE LATE bonus to the final ranking score. However, the baseline predictor can still underestimate analogues that carry favorable AC-enriched fragments, particularly near steep SAR regions. To address this, we introduce CAFE LATE, a lightweight post hoc selection-stage extension of CAFE. Whereas CAFE operates during fragment prioritization by extracting enrichment signal from AC pairs, CAFE LATE reuses this supervised fragment knowledge during candidate ranking by assigning an additional bonus to newly generated molecules containing positively enriched AC fragments.

For each newly synthesized molecule $m$, it checks whether each AC fragment $f$ occurs in $m$ using RDKit substructure match of the normalized fragment pattern (same key used by CAFE). Presence is encoded by the indicator:

20 $${\delta}_{f}(m)=1\{f\subseteq m\}$$

Multiple occurrences of the same fragment $f$ in molecule $m$ are counted once (prevents length bias from repeated substructures). CAFE LATE converts fragment level evidence into a single signed bonus by summing the previously computed $E(f)$ over fragments found in *m*:

21 $$B(m)=\sum_{f\in {f}_{AC}}{\delta}_{f}(m)E(f)$$

AC positive motifs ($E\left(f\right)>0$) increase the ranking score through trough $B(m$*),* and molecules containing no positively enriched CAFE fragments have $B(m)=0$ and therefore remain unchanged by CAFE LATE.

**Fig. 2:**
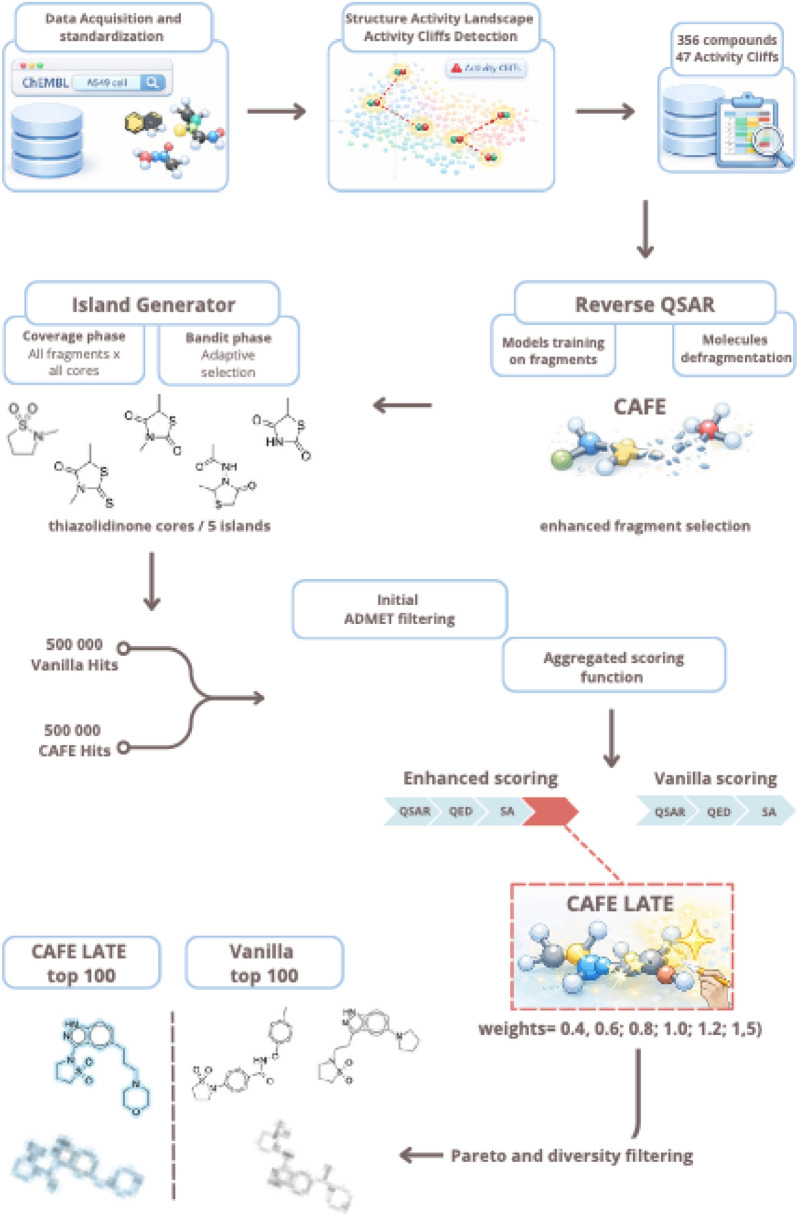
Overview of the AC-aware de novo design workflow, with simplified depiction of CAFE and CAFE LATE intervention

Final selection proceeds in two parallel arms. In the vanilla arm, used here to denote the baseline workflow without AC-derived post hoc boosting, we set λ = 0 and select the best 100 compounds by ranked aggregated score $\varphi \left(m\right)$. In the CAFE arm we run the same pipeline repeatedly over a grid of ${\lambda}_{CAFE}=\{0.4;0.6;0.8;1.0;1.5\}$ values, each time independently producing the best 100 hits ranked by $\varphi \left(m\right)$. Final selection follows a multi-stage funnel that guarantees quality and diversity while always filling to the target size. We first pre-rank candidates by $\varphi \left(m\right)$ and retain a working pool. We then compute a Pareto front on the three base objectives QSAR, SA′ and QED to keep only non-dominated solutions. The front is re-ranked by $\varphi \left(m\right)$, after which we apply diversity selection with ECFP4 (radius 2, 2048 bits) and Tanimoto similarity using a greedy best-first procedure that starts from the highest-scoring molecule and iteratively admits the next most dissimilar candidate. If diversity constraints make the set too small the similarity threshold is relaxed stepwise until the target count is reached. A minimum representation per core scaffold is enforced, canonical SMILES de-duplication is applied, and ties are broken by $\varphi \left(m\right)$. Each ablation arm yields exactly 100 molecules. For discovery-oriented analyses we additionally build a union of all unique candidates from CAFE boosted runs, which is used downstream to surface “all-star” candidates irrespective of the specific weight that promoted them. The simplified scheme of new molecules generator with enhanced AC-aware methods CAFE and CAFE LATE is presented on Fig. 2.

### Molecular docking

Protein structures were retrieved from the Protein Data Bank (PDB) [56], identified by their PDB IDs (1M17, 4I23, 8GUA, 4EKL, 5OQ4, 4LMN, 5HD4, 6SCM, 5EHR, 4L7B, 5L2S, 5L2I, 5L2T), stored locally and preprocessed to remove alternate conformations, water molecules, ions, and crystallization additives. Hydrogen atoms were added, and protonation states were assigned using pdbfixer [57]. Cleaned receptor structures were converted to PDBQT format using AutoDockTools [58]. Ligand structures, provided as SMILES representations, were transformed into 3D conformations using Open Babel [59] and converted to PDBQT format. Ligand protonation states were assigned during 3D preparation using the standard Open Babel workflow under consistent, near-physiological conditions across the full ligand set.

The docking grids were automatically generated based on native ligand coordinates. The grid box was defined using the ligand center of mass with a padding of 4 Å to ensure the binding site was fully encompassed [60]. Docking simulations were conducted using Smina, an optimized fork of AutoDock Vina [61], using a fixed protocol across all receptors and ligands. Parameters were set to default values, including exhaustiveness of 24 and 10 docking poses per ligand-receptor pair. Native ligand re-docking served as validation, with the root mean square deviation (RMSD) calculated to assess docking accuracy. An RMSD ≤ 2.0 Å was considered a successful redocking [62].

Docking poses were evaluated based on calculated binding affinities (kcal/mol). For downstream comparative analyses, the retained pose for each ligand–receptor pair was defined as the lowest-energy valid pose returned under the same docking setup. Docking scores were used as a comparative prioritization criterion within this fixed protocol rather than as absolute estimates of binding affinity.

Ligands with docking scores more favorable than those of the corresponding native ligands under the same protocol were selected as preliminary hits for further refinement. Interaction profiles of the best docking poses were analyzed using the Protein–Ligand Interaction Profiler (PLIP) [63], generating detailed reports of interaction types such as hydrogen bonds, hydrophobic interactions, and π-stacking. Complex structures (receptor-ligand) of the top hits were visualized using py3Dmol [64] within Jupyter Notebooks and 2D interaction diagrams were created using python scripts. Visualizations included highlighting binding sites, ligand conformations, and receptor-ligand interactions.

### Ablation analysis

To quantify the contribution of CAFE/CAFE LATE beyond baseline QSAR scoring, we dock, per receptor, the top 100 compounds from vanilla (baseline) arm and each CAFE top 100 (for every ${\lambda}_{CAFE}$) under the same protocol, filter invalid poses (non-negative scores) and native ligands, and then form one-to-one matched pairs within each target by rank with a molecular-weight tolerance of ≤ 25 Da (unmatched ranks are discarded to preserve comparability). For every retained pair we compute:

20 $$\Delta {\rm E}={E}_{CAFE}-{E}_{VAN}$$

on minimal binding affinities, so that $\Delta {\rm E}<0$ denotes a CAFE LATE candidate win (lower binding affinity), and we summarize per-target and pooled effects using the paired Wilcoxon signed-rank test on $\Delta {\rm E}$ with two-sided p-values and the matched-pairs rank-biserial correlation as effect size, alongside outcome-focused statistics that reflect medicinal-chemistry priorities: namely the mean $\Delta {\rm E}$, the 5th-percentile improvement (Q5) capturing uplift among the very best candidates, and the 25th-percentile (Q25) for the top quartile. Finally, across CAFE LATE weights we identify the “best” configuration by the most negative mean Q5 averaged over receptors (ties broken by mean $\Delta {\rm E}$), use this single configuration for head-to-head top-k visualizations against vanilla (ranks 1–6 per receptor), and report the unified CAFE union only for discovery summaries

### Molecular dynamics

Based on the molecular docking results, both native ligand complexes and ligands exhibiting superior predicted binding affinities compared to their native counterparts (*better-than-native* hits) were selected for further dynamic evaluation. In total, molecular dynamics (MD) simulations were performed for approximately 245 protein–ligand complexes, enabling a systematic assessment of binding stability and interaction persistence beyond static docking poses. As a result, four protein structures were used in the MD campaign and downstream analysis: 1M17, 4I23, 5L2S, 5OQ4.

All MD simulations were carried out using OpenMM 8.2.0 [65]. Prior to simulation, receptor structures were carefully repaired to ensure structural completeness and consistency. Missing residues and atoms were reconstructed, non-standard residues were standardized, and protonation states were preserved, using PDBFixer. Ligand topologies and parameters were generated separately to ensure compatibility with the selected force fields.

Protein atoms were described using the Amber ff14SB force field [66], while ligands were parameterized with GAFF-2.11 [67]. Each protein–ligand complex was solvated in an explicit TIP3P water box with a minimum padding of 10.0 Å from the solute to the box edge. Physiological conditions were mimicked by adding Na⁺ and Cl⁻ ions to achieve a final salt concentration of 150 mM.

A multistage equilibration protocol was applied to all systems prior to production simulations. This protocol consisted of energy minimization to remove steric clashes, gradual heating from 50 to 300 K, progressive release of backbone restraints, and equilibration under constant volume (NVT), followed by constant pressure (NPT) conditions.

Temperature and pressure were controlled using Langevin dynamics and a Monte Carlo barostat, respectively, while long-range electrostatics were treated with the Particle Mesh Ewald (PME) method. Following equilibration, each complex was subjected to a 100 ns production MD simulation at 300 K. A timestep of 1 fs was employed, and trajectory frames were saved every 100 fs for subsequent analysis. This extensive MD campaign enabled comparative evaluation of native versus better-than-native ligands in terms of structural stability, interaction longevity, and dynamic behavior within the binding site.

To quantify stability and pose retention, we computed Ligand RMSD (only heavy atoms), after fitting to the protein backbone; reported as time series, Pocket RMSD (residues within a defined radius around the ligand within 6 Å) to capture local receptor stability. Moreover, RMSF per residue, with emphasis on binding-site residues (e.g., residues within 6 Å of the ligand in the starting structure) to measure binding-site flexibility and Ligand–pocket COM distance and a bound-state fraction, defined by a geometric criterion, to detect partial unbinding or large rearrangements.

## Results and discussion

### Chemical space and structure activity landscape

After standardization, de-duplication, and scope filtering, the operational thiazolidinone dataset consisted of 356 compounds (Additional file 1), with a workable potency class balance: 117 actives (32.9%) and 239 inactives (67.1%). We removed a small set of “odd compounds” bearing cholesterol derived motifs (Additional file 2), because they lie outside thiazolidinone area of the chemical space and would inject nontransferable signal for small-molecule optimization. Using ECFP4/Tanimoto similarity $\uptau$ and a log-scale potency difference (${\Delta pIC}_{50}$), we identified 47 AC pairs (Additional file 3), covering 54 unique molecules, meaning 15.2% of the dataset participate in at least one AC pair. Within all high-similarity neighborhoods we observe: 349 pairs with $\uptau$ ≥ 0.80 (of which 13.5% are AC), 119 pairs with $\uptau$ ≥ 0.85 (10.1% AC), and 18 pairs with $\uptau$ ≥ 0.90 (5.6% AC), indicating that while most close neighbors behave smoothly, a non-trivial fraction encodes steep SAR.

**Fig. 3:**
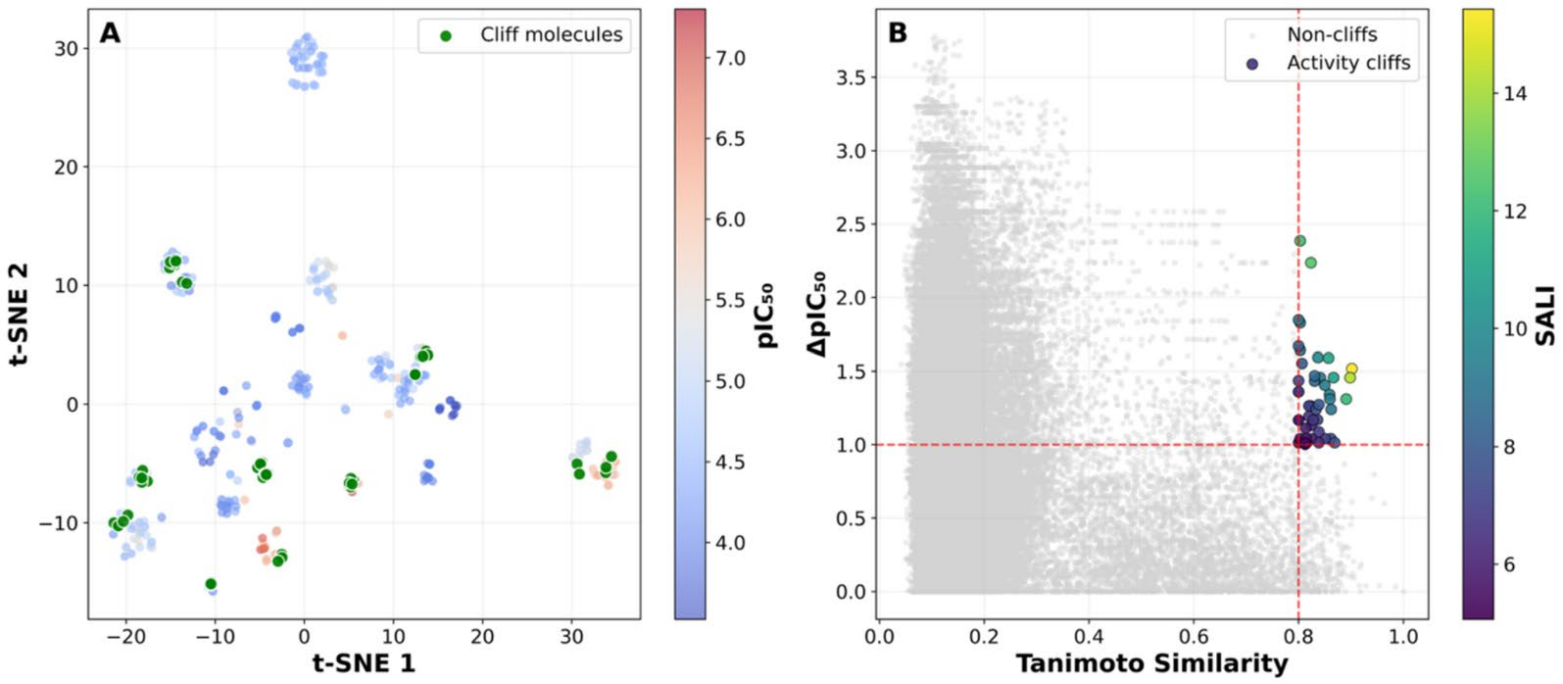
**A** t-SNE projection of the thiazolidinone chemical space, computed on ECFP/Tanimoto, colored by pIC_50_ values. Green scatters are AC molecules. **B** Distribution of $SALI$ values across molecule pairs in thiazolidinone dataset

To visualize thiazolidinones chemical space, we conducted a t-SNE projection of ECFP4 space (Fig. 3A), which reveals molecules gathered in dense scaffold consistent clusters with broadly coherent potency, concentrating easy to learn SAR signal. Critically, AC pairs are mostly located on the edges of those smooth regions, where minor structural edits yield disproportionate activity changes. This is precisely where disrupted signal mis-calibrates model, leading to overgeneralization and bad predictions. Those observations are only unpowered by MDS projection showing structurally similar compounds aggregated together while AC are randomly scattered among whole chemical space leading to SAR discontinuation (see Figure S1).

To quantify those discontinuities, hidden in clusters, we computed $SALI$ which captures how different a pair of seemingly similar compounds is and set it against structural similarity to produce a SAS map. As a result, we detected twelve AC pairs with $SALI$ $\ge$ 10, forming compact “hot zones” of very steep SAR. Of these, 7 pairs also meet our AC rule (${\Delta pIC}_{50}$ ≥ 1.0 and $\uptau$ ≥ 0.8) (Fig. 3B). The remaining 5 are $SALI$ only steep pairs where extreme similarity magnifies moderate ${\Delta pIC}_{50}$, still mechanistically informative, and easy to miss with a binary AC definition. Together, t-SNE, MDS and the SAS map provide complementary views of the landscape: rule-based AC emphasize large ${\Delta pIC}_{50}$ among close neighbors, while $SALI$ also surfaces micro-edits that create sharp local gradient. Practically, the dataset offers favorable mix for model guided design: cluster structured regions support generalization, and exposed AC carry valuable insights about crucial structural edits driving activity. In the next section we convert these high-gradient signals into supervised fragment knowledge for r-QSAR and generation, so that the library prioritizes thiazolidinone variants carrying the key modifications linked to activity gains.

### Fragments library

**Fig. 4:**
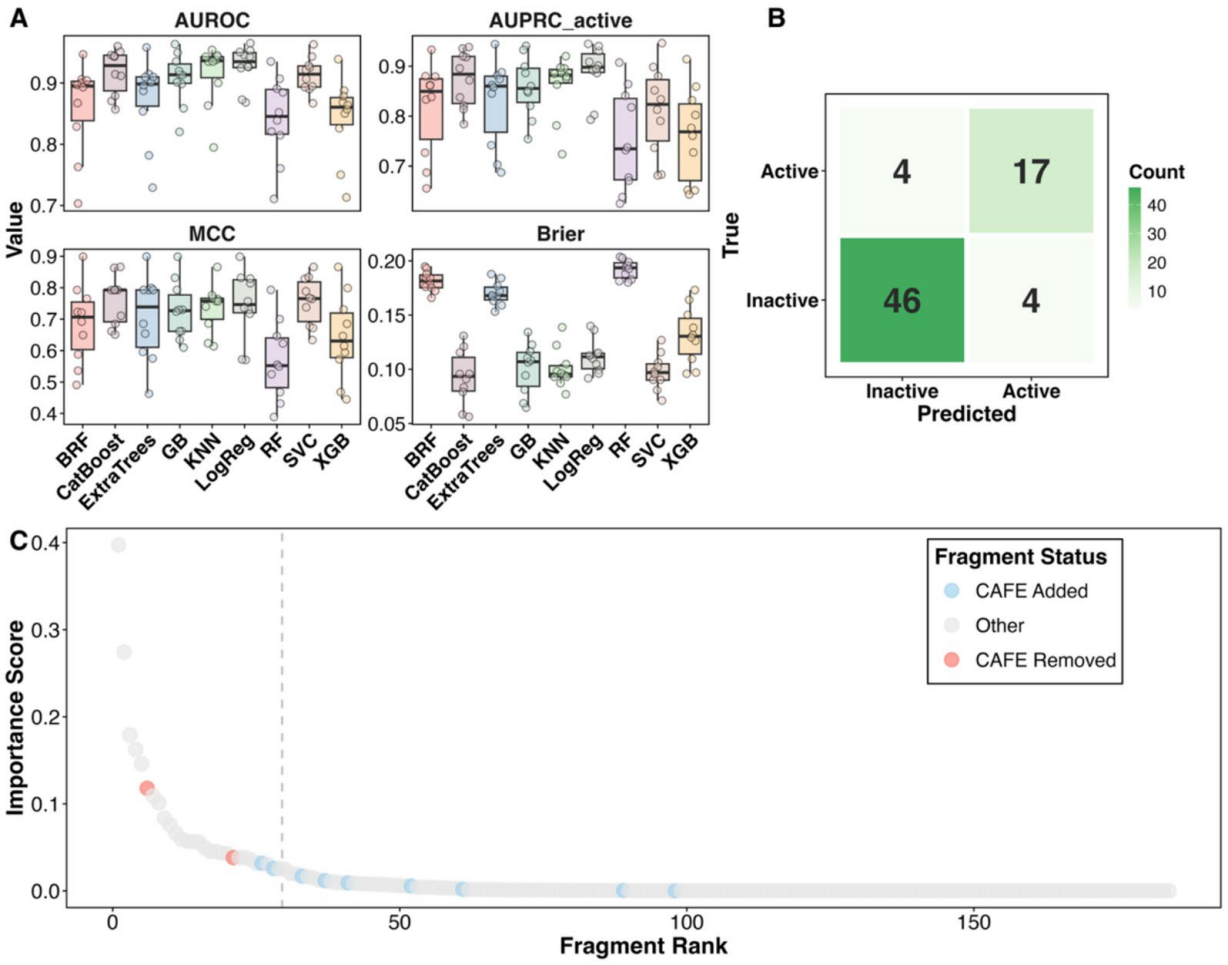
The scatter plot with error bars of r-QSAR performance trained on nine ML engines **A**. The confusion matrix of the best r-QSAR model- logistic regression **B**. The fragments ranking by the models’ importance score (**C**)

Across trained ML engines, using the area under the precision–recall curve for the active class (AUPRC_active_) as the primary criterion, logistic regression delivered the best mean performance of 0.889 (95% CI 0.856–0.917), and was therefore selected as the r-QSAR architecture (Fig. 3A, detailed metrics in Table S.1). The model misclassified 8 compounds (4 false negatives and 4 false positives), as shown on confusion matrix (Fig. 4B) these errors concentrate near high-gradient regions where small structural edits produce large potency flips, outside those zones, interpolation is stable and the classifier behaves as expected. This pattern justifies our choice of AUPRC_active_ (robust to prevalence and aligned with the practical objective of recovering actives) and motivates a fragment level audit of failures. To identify and extract fragments responsible for those failures, we implemented a new logic CAFE, which corrects model choices by injecting important SAR signal hidden in AC pairs. Consistent with that, vanilla r-QSAR (without CAFE interference) identified 25 fragments with importance score cutoff as the most influential on compound’s activity. However, as depicted on Fig. 4C, CAFE analysis revealed that r-QSAR overlooked nine important activity driving fragments, and critically included two fragments that are likely to occur on the inactive side of AC. Those falsely recognized motifs explain models’ failures and metrics dropouts. If used as building blocks, they would bias generation toward suboptimal proposals. The intervention is surgical and keeps the library consistent with the measured landscape: smooth SAR regions still support generalization, while high-gradient edits are now explicitly represented as constructive design rules rather than noise. The final fragments library modified by CAFE (Table S.2), gives promise for generation of more reliable proposals.

### Hit discovery

**Fig. 5:**
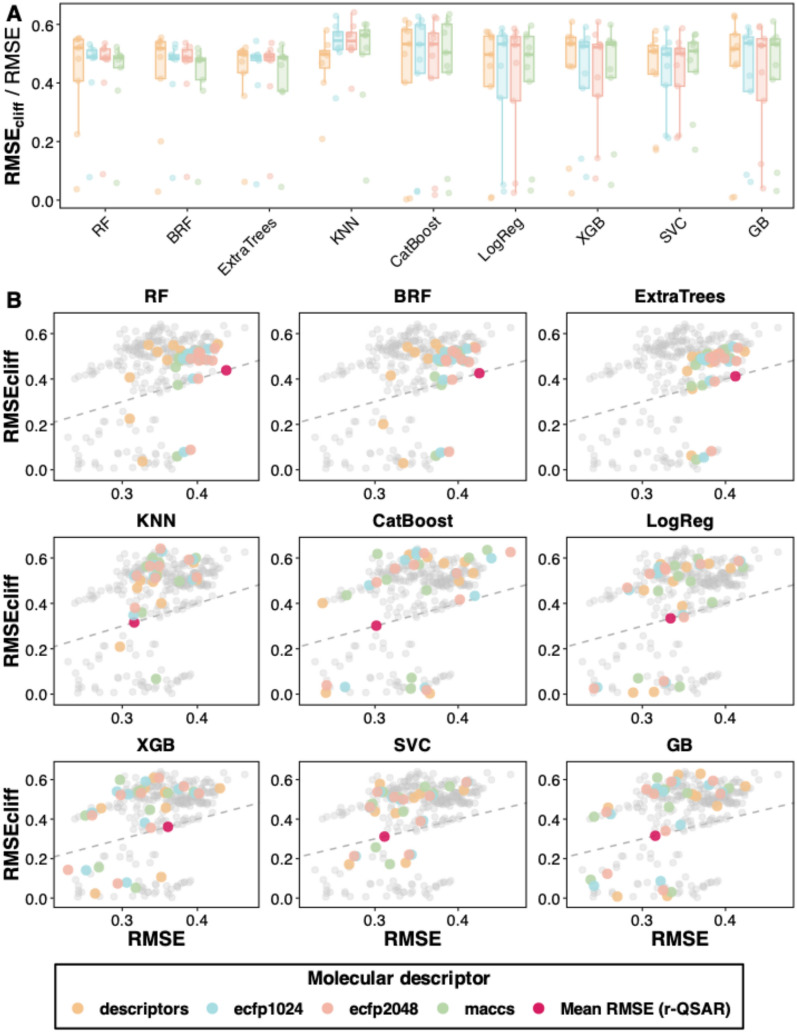
**A** The ${RMSE}_{cliff}$ values of QSAR models and **B** comparison of ${RMSE}_{cliff}$ values against global RMSE computed on several molecular representations from ten independent training splits

De novo generated thiazolidinone candidates must be preliminary assessed for predicted activity against the A549 lung cancer cell line, to prioritize productive design and by filtering out suboptimal building blocks combinations. For this task, we follow a MoleculeACE guideline and select the best prediction model based on AC-aware metric (${RMSE}_{cliff}$), instead of global ones that could show overoptimistic results when tested on non-AC molecules. We used our version of that metric tuned for the classification task specificity, which identified BRF trained on MACCS as the most AC-aware model with the most robust results of ${RMSE}_{cliff}$= 0.421, 95% CI 0.324–0.487 (see Fig. 5A and Table S3 for detailed performance metrics).

**Fig. 6:**
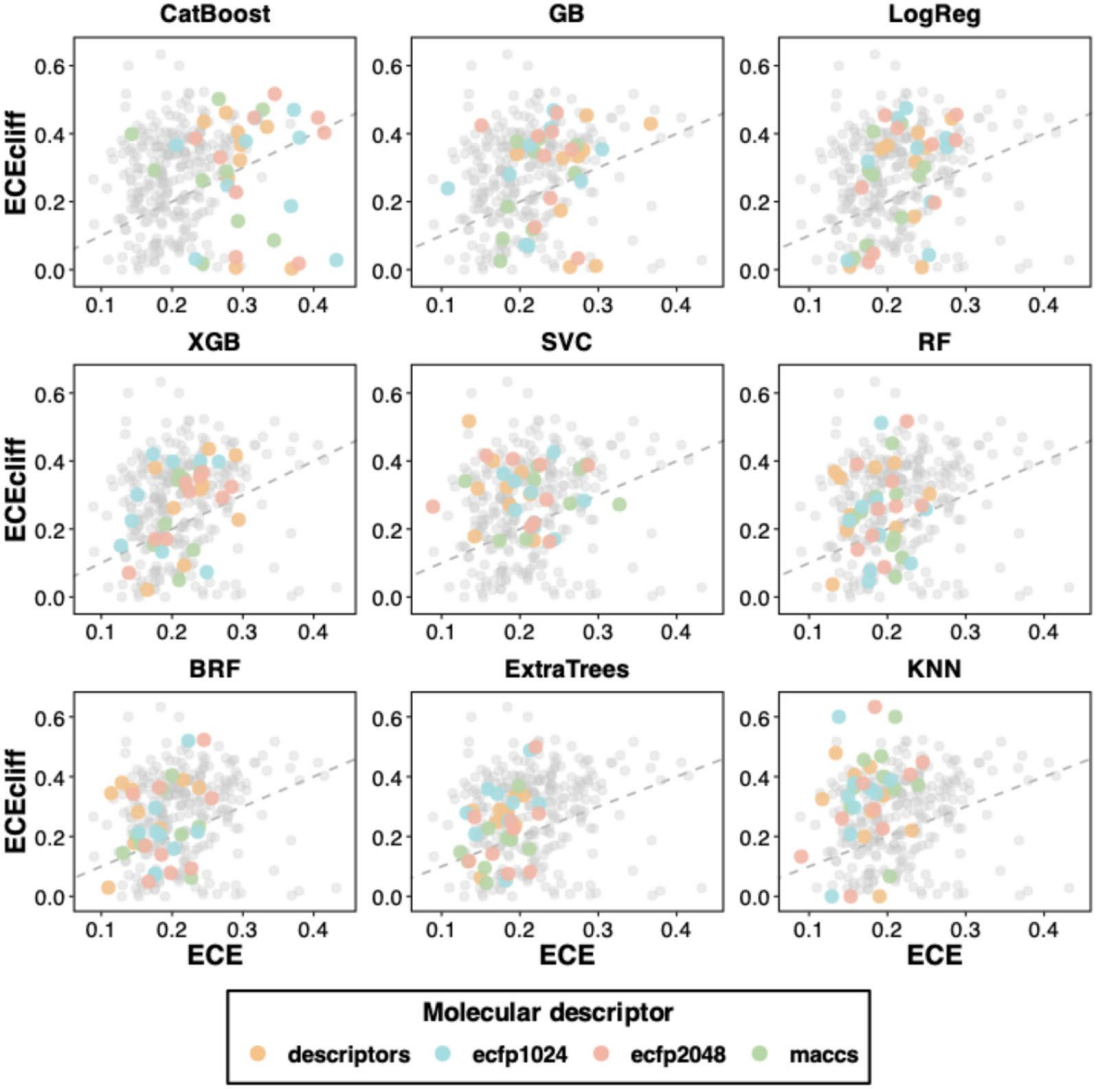
Model calibration across evaluated QSAR configurations, assessed using global Expected Calibration Error (ECE) and AC restricted ECE (ECE_cliff_)

This model choice is further substantiated by the broader error and calibration patterns observed across QSAR settings. Global RMSE is frequently lower than ${RMSE}_{cliff}$, indicating that performance estimated over the random test set is often more optimistic than performance measured in steep SAR regions. In Fig. 5B, many model configurations lie above the dashed identity line, confirming that prediction error on AC compounds is systematically higher than the corresponding global error. This pattern highlights the extent to which AC distort the apparent predictive performance of QSAR models and demonstrates that global error metrics alone can underestimate the true difficulty of the task. Importantly, the pink points representing mean global RMSE calculated on the AC-free test sets used in the r-QSAR setting are, in most cases, shifted toward even lower values, further illustrating how removal of AC from the evaluation set creates an artificially easier and more favorable assessment scenario. The same trend is reflected in the calibration analysis (Fig. 6). For most QSAR settings and across the majority of split realizations, AC restricted expected calibration error (ECE_cliff_) exceeds the corresponding global ECE, again with many points located above the dashed line. Thus, activity cliffs impair not only predictive accuracy, but also the reliability of probabilistic outputs. Taken together, these results show that AC define a region of chemical space in which both error magnitude and probability calibration deteriorate, making global metrics insufficiently informative for model selection. In this context, the use of ${RMSE}_{cliff}$ as the primary criterion is not only methodologically justified, but necessary to identify a model that remains robust in the most challenging and decision-relevant part of the SAR landscape.

Having established a CAFE-corrected fragment library and selected the best-performing QSAR scorer, we next deployed both components in the de novo design stage. At the same time, we remained aware that even the most AC-robust available classifier, selected here through ${RMSE}_{cliff}$ cannot fully resolve the intrinsic predictive difficulty associated with steep SAR regions. Because learning activity directly from chemical structure becomes particularly challenging in the vicinity of AC, we introduced CAFE LATE as a supervised post hoc correction layer that adjusts the QSAR-based ranking of newly generated candidates carrying positively enriched CAFE fragments.

Generation was therefore conducted in two parallel arms. The first was the vanilla branch, corresponding to the baseline workflow without AC-derived post hoc correction. The second was the AC-aware branch, in which the corrected fragment library was combined with CAFE LATE supervision during candidate ranking. To determine the most effective contribution of the AC-aware bonus while preserving balanced exploration of chemical space, we evaluated a grid of post hoc boosting weights, ${\lambda}_{CAFE}=\{0.4;0.6;0.8;1.0;1.5\}$, applied independently during candidate scoring.

Because the final fragment library contained five distinct thiazolidinone ring variants, we adopted an island-style genetic generator to distribute exploration more evenly across the scaffold space. In both branches, 500,000 candidates were generated and subsequently standardized and evaluated using the aggregated score $\varphi (m)$, composed of the QSAR-predicted probability, synthetic accessibility, and drug-likeness scores. In the AC-aware branch, this baseline score was further corrected by the CAFE LATE bonus under the tested ${\lambda}_{CAFE}$ settings. As a result, we obtained one top100 set from the vanilla branch and one top100 set from each AC-aware weight setting, giving six top100 datasets in total. The overlap between the vanilla and CAFE LATE top-ranked sets decreased progressively with increasing ${\lambda}_{CAFE}$, reaching 5%, 1%, and finally 0% for the strongest boosting settings. In total, 282 unique candidates were identified and forwarded to molecular modeling studies (see Additional file 4).

### Mechanism supported lead selection

To confirm results from preliminary assessment and choose the best candidates we implemented mechanistic prediction studies and compared the docking scores of all unique 282 candidates against those of the corresponding native ligands across well-characterized protein targets relevant to A549 cancer cell line. Those included:

- 1M17, Epidermal Growth Factor Receptor (EGFR) wild kinase domain co-crystallized with gefitinib a reversible, ATP-site, type-I binder that blocks receptor autophosphorylation. In A549, upstream receptor inhibition may be partially bypassed by constitutively active KRAS signaling [68].
- 4I23, EGFR wild kinase domain co-crystallized with dacomitinib, clinically used inhibitor [69].
- 8GUA, phosphoinositide 3-kinase alpha (PI3Kα, E542K mutant) with alpelisib (BYL-719). Direct structural reference for the class-I PI3Kα pocket engaged by an approved α-selective inhibitor [70].
- 4EKL, AKT1 co-crystallized with the clinical ATP-site inhibitor ipatasertib (GDC-0068). Supports docking and pharmacophore transfer for the central PI3k and AKT node [71].
- 5OQ4, class I PI3K/mTOR dual inhibitor (PQR309/paxalisib) in complex with PI3K. Relevant for KRAS mutant cancers [72].
- 4LMN, MEK1 bound to cobimetinib (allosteric, type-III site). Defines the MEK1 allosteric pocket and activation-loop geometry; essential for modeling the RAS → RAF → MEK → ERK cascade that sits immediately downstream of KRAS in A549 [73].
- 5HD4, ERK2 co-crystallized with SCH772984 (inhibitor induced pocket). Shows the distinctive inhibitor induced binding mode in ERK. Valuable when probing terminal MAPK pathway blockade after KRAS/MEK [74].
- 6SCM, SOS1 in complex with BI-3406 (protein–protein interaction blocker). Orthosteric disruption of the SOS1–KRAS nucleotide exchange interface. Structurally and mechanistically aligned to dampening KRAS activation irrespective of the specific codon-12 substitution in A549 [75].
- 5EHR, SHP2 (PTPN11) co-crystallized with the allosteric inhibitor SHP099. Stabilizes SHP2 in an autoinhibited conformation at the interface of its SH2 and PTP domains. Structurally underpins strategies that blunt receptor-tyrosine-kinase-RAS signaling input feeding the KRAS pathway[74].
- 4L7B, KEAP1 Kelch domain bound to a small-molecule antagonist. High-resolution Kelch-propeller pocket usable for modeling disruption of KEAP1-NRF2. Relevant because A549 carries KEAP1 loss-of-function and exhibits NRF2 pathway dysregulation [76].
- L2S, CDK6 co-crystallized with abemaciclib, 5L2I with palbociclib, and 5L2T with ribociclib. Three orthogonal, drug-resolved views of the CDK6 ATP site that support modeling cytostatic control over G1/S progression. Useful phenotypic leverage even when signaling is KRAS dominant [77].

**Fig. 7:**
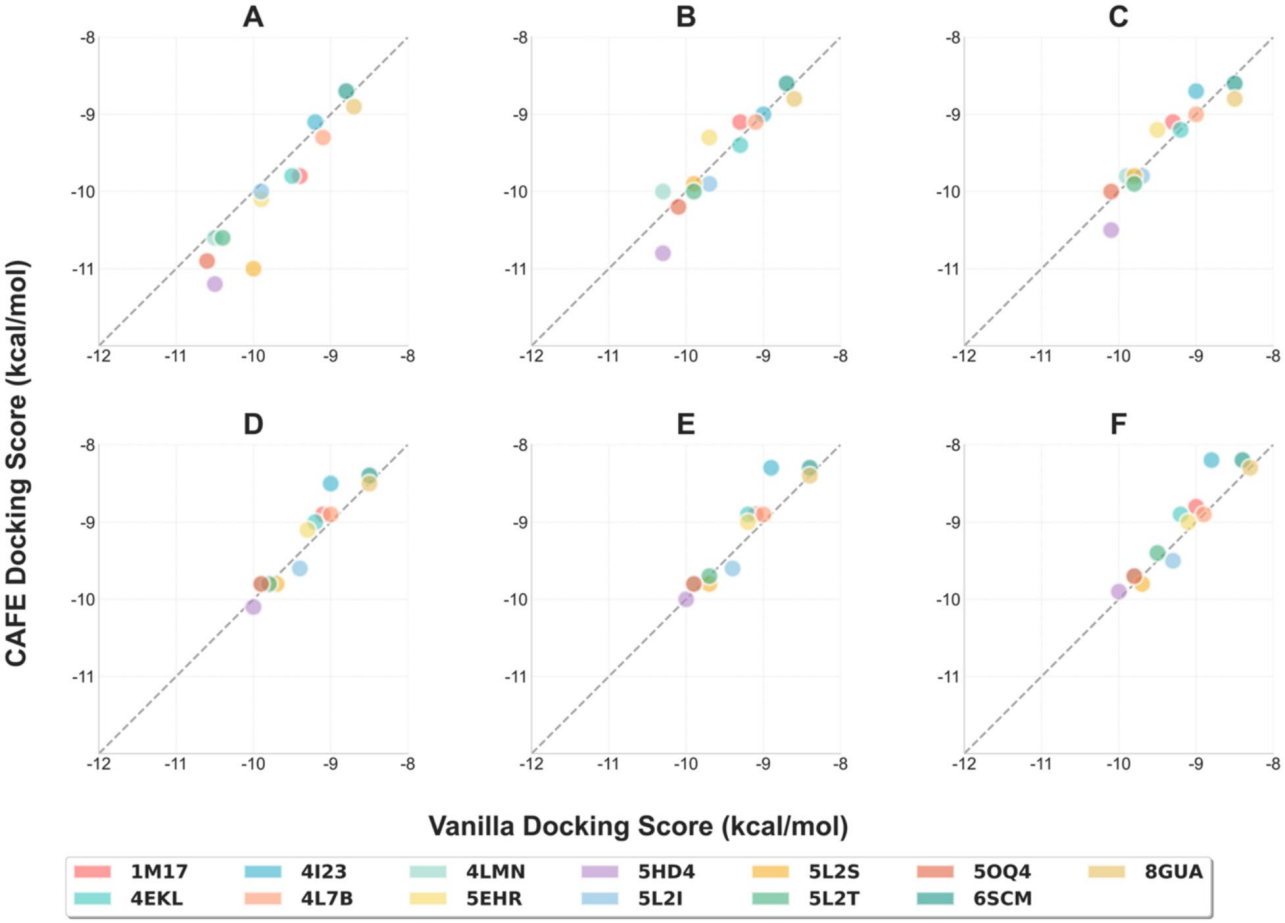
Comparison of top 6 ranked molecules between CAFE and vanilla approaches across 13 molecular targets (**A**–**F** corresponding to 1–6 ranked hits, respectively)

All native ligands were successfully redocked into their cognate receptors, and the resulting poses matched the crystallographic references with RMSD ≤ 2.0 Å (Table S4), qualifying the receptor ensemble and docking setup. Preliminary analysis revealed influence of CAFE LATE on designing candidates with more favorable docking scores against many of studied molecular targets. The largest mean paired improvement was observed for the 5EHR receptor in the ${\lambda}_{CAFE}$= 0.6 dataset (Δ =  − 0.139 kcal/mol across 95 matched pairs; Wilcoxon signed-rank test, *p* < 0.0001). Although differences of this magnitude should not be overinterpreted in absolute scoring terms, their directionality was consistent under the same docking protocol and therefore remains informative as a comparative signal. Importantly, the effect of AC-aware boosting became more pronounced at the top end of the ranking, which is the practically relevant region for hit prioritization. For instance, the mean improvements in binding affinities of top 5 matched pairs of ${\lambda}_{CAFE}=0.8$ and vanilla dataset for 5EHR, 4I23, 4L7B were -2.5 kcal/mol, -0.980 kcal/mol and -0.7 kcal/mol, respectively. As depicted on Fig. 7 in head-to-head comparison of top 6 docking results from ${\lambda}_{CAFE}=0.8$ dataset to vanilla, hits generated with AC boosting achieved better predicted binding affinities for most targets (points below dashed line symbolize CAFE LATE candidate with more favorable binding affinity colored by protein target).

Across the 282 unique combined CAFE LATE and vanilla hit molecules docked against all thirteen validated protein structures, 241 protein–ligand complexes achieved docking scores more favorable than the corresponding native ligand (see Additional file 5). Among those, 63.9% originated from CAFE LATE arm, indicating that vanilla generation model would miss more than a half ligand–protein complexes beating referent results, highly decreasing a chance for in vitro success. The best three docking scores in better-than-native dataset were formed by CAFE LATE candidates (−11.0 | −9.7; −10.9 | −8.2; −10.8 | −8.8 kcal/mol). Most importantly, five candidates showed better results than native ligands for more than one molecular target suggesting a potential multitargeted activity, and four from them were designed with AC-aware modeling strategy. For illustration, molecule **314** (from dataset with ${\lambda}_{CAFE}=0.6$) formed a particularly strong complexes with 1M17: min|max scores of −9.8 | −7.4 kcal/mol, 5OQ4: −9.8 | −7.3 kcal/mol, and 5L2S: −11.0 | −9.7 kcal/mol, compared to the native ligands results of −7.1 | −6.4; −9.1 | −8.1; −10.2 | −8.9, respectively.

All complexes from better-than-native dataset were transferred to structure-based comparative refinement: short replicate molecular dynamics (MD) simulations to quantify pose stability and interaction persistence, followed by MM-GBSA postprocessing to estimate relative binding free energies from MD snapshots. To reduce receptor conformation bias, per-ligand results were aggregated across the validated receptor ensemble (robust statistics over the best conformations), and a consensus ranking integrated post-MD MM-PBSA, pose stability, and contact occupancy.

We evaluated the proposed CAFE LATE molecule design approach against a vanilla baseline using MD simulations across four protein–ligand complexes from better-than-native molecular docking analysis: 1M17 (n = 203 total systems), 5OQ4 (n = 23), 4I23 (n = 16), and 5L2S (n = 3). For each complex, the native ligand was re-docked and simulated under the same protocol and used as a per-target reference. For each designed ligand we computed: Pocket RMSD, Pocket RMSF, Ligand RMSD, Ligand RMSF, and Binding Energy. The whole distribution of results is presented on Figure S2.

In the cross-section by targets where we have both methods (1M17, 5OQ4, and 4I23) we run 91 vanilla ligands and 148 generated by the proposed algorithm. CAFE LATE shifts the distributions towards better structural stability more often than the baseline vanilla approach, which is best quantified as the percentage of candidates better than native one on each metric. Pooled across proteins, CAFE LATE molecules win more often on Pocket RMSD, Pocket RMSF, Ligand RMSD and Ligand RMSF, as shown in Table 1.

**Table 1:** Summary of molecular dynamics simulation metrics for studied protein—ligand complexes

Metric	CAFE LATE molecules			Vanilla molecules		
	Better	Worse	%	Better	Worse	%
Pocket RMSD	95	54	**63.76**	55	37	59.78
Pocket RMSF	41	108	**27.52**	21	71	22.83
Ligand RMSD	70	79	**46.98**	37	55	40.22
Ligand RMSF	80	69	**53.69**	43	49	46.74
Binding Energy	55	94	36.91	41	51	**44.57**

In MD MM-PBSA alone, the picture is more protein-dependent and does not show a unidirectional advantage of CAFE LATE across the entire set, suggesting that the main strength of CAFE LATE is revealed in stability metrics, while binding energy is more sensitive to the nature of the pocket and the underlying target difficulty. The largest and most representative set is 1M17 (203 systems in total: 73 vanilla, 129 CAFE LATE, and native), so we treat it as the main test. In 1M17, CAFE LATE consistently increases the percentage of candidates more stable than native in both ligand and pocket metrics, especially for Ligand RMSD and Ligand RMSF, as well as others. For MM-PBSA in 1M17, both methods achieve a comparable, moderate rate of outperforming native one. The key point, however, is that despite the moderate “average” advantage on MM-PBSA, CAFE LATE delivers strong individual hits that simultaneously satisfy all stability and energy criteria. In 1M17, CAFE LATE candidates such as **239** or **343** improved on all metrics relative to native, while in the vanilla set, similar cases are rarer.

**Fig. 8:**
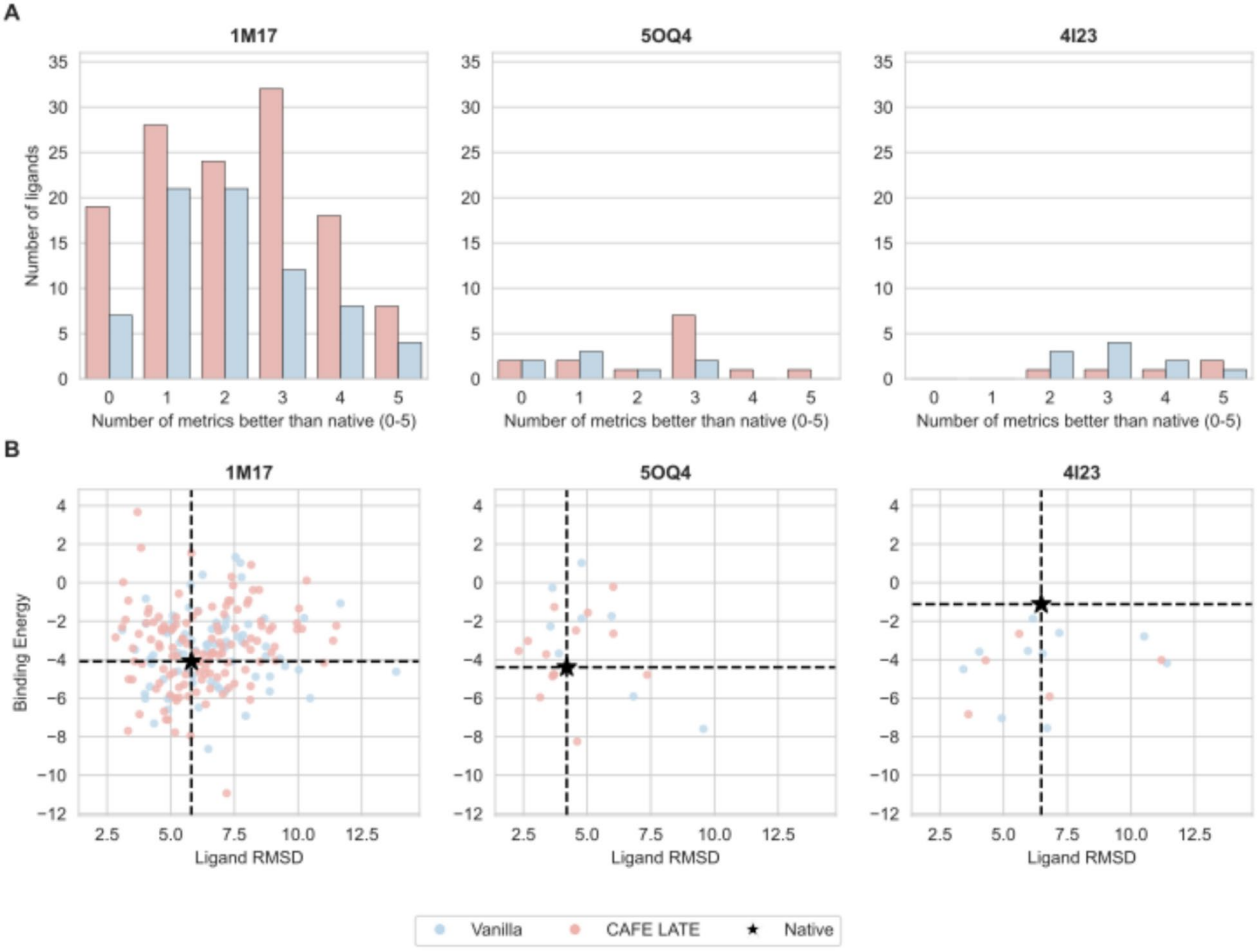
**A** Multi-metric improvement profile relative to the native ligand. For each protein, we show the distribution of the number of molecular dynamics metrics for which a given ligand outperforms the native reference for each protein target. **B** Stability and energy tradeoff for ligand binding across protein complexes. Scatter plots show Ligand RMSD versus Binding Energy for ligands generated by the vanilla approach and by the CAFE method for each protein complex

To show whether the method produces individual hot candidates or rather a general shift in the distribution we present Fig. 8A, where the mean of the number of metrics improved simultaneously is higher for CAFE LATE (2.20 vs 2.07), reflecting a subtle but systematic shift towards multimetric improvement.

In 5OQ4 (23 complexes: 8 vanilla, 14 CAFE LATE, and native one), the advantage of AC-aware candidates is more pronounced and covers both stability and energy. CAFE LATE molecules more often improve Pocket RMSF (42.9% vs. 12.5%), Ligand RMSD (57.1% vs. 37.5%), Ligand RMSF (78.6% vs. 50.0%), and Binding Energy (42.9% vs. 25.0%), which indicates that the CAFE LATE algorithm better targets stable poses while maintaining or enhancing energetic interactions. The stability vs energy tradeoff is particularly well illustrated by the Pareto analysis shown in Fig. 8B. In this representation. The quadrant corresponding to simultaneously improved stability and binding energy (lower RMSD and Binding Energy) provides an immediate visual summary of method performance. In this region, ligands generated by CAFE are markedly more abundant than those obtained with the vanilla approach. Notably, ligand **241** exemplifies this behavior by outperforming the native reference across all five evaluated metrics, including a substantially improved Binding Energy value of − 5.951. The whole calculation metrics are available in Additional file 6.

**Fig. 9:**
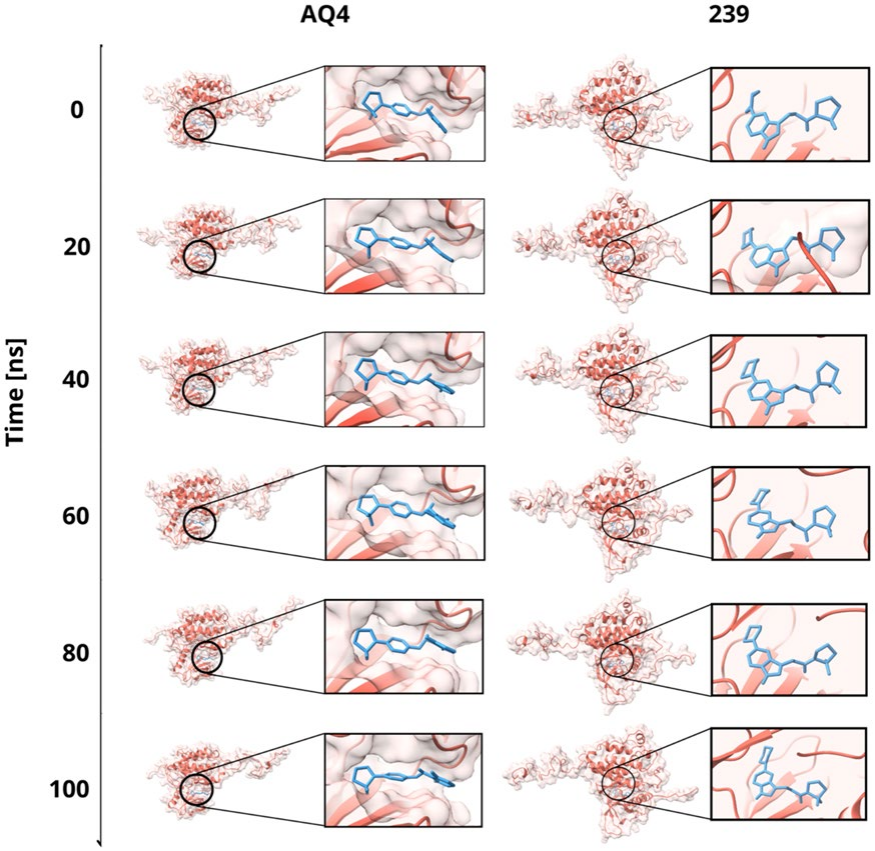
Visualization of head-to-head comparison of protein–ligand complexes (1M17 with native vs the best generated compound 239) using MD simulation in 100 ns

To identify the most promising candidates generated in this study, we ranked all non-native ligands across protein targets according to their multi-metric improvement relative to the native redocked reference. The five highest-ranked ligands’ complexes all outperform the native reference simultaneously across all five evaluated metrics, representing the strongest overall candidates identified in this benchmark shown in Table 2. Notably, 4 out of the 5 top ligands were generated by the CAFE LATE method, despite the presence of strong vanilla candidates, indicating that our algorithm not only increases the overall hit rate but also enriches the good quality region of chemical space. As depicted on Fig. 9, the best overall results were observed for hit 239 with computed binding energy of −7.78 kcal/mol formed with 1M17 target (compared to native ligand result of −4.09 kcal/mol), what complemented stability of favorable protein ligand interactions previously detected in molecular docking simulations (see Figure S3).

**Table 2:** Top-performing ligands across molecular dynamics benchmark identified against 1M17 protein target

Ligand ID	Ligand structure		Method	Binding energy [kcal/mol]	QED | SA
239			CAFE LATE	−7.78	0.85 | 0.77
343			CAFE LATE	−7.70	0.72 | 0.81
86			Vanilla baseline	−7.31	0.87 | 0.81
265			CAFE LATE	−7.12	0.80 | 0.78
271		CAFE LATE		−6.84	0.87 | 0.82

Fragments extracted by AC-aware method CAFE are highlighted in color blue

All molecules show 5/5 improved metrics compared to native ligand results

To place these hits in the context of the modeled chemical space, we additionally identified their nearest analogs in the training set using the same ECFP4/Tanimoto definition applied throughout the study. The resulting similarities were moderate (0.338–0.537; Additional file 7), indicating that the top-ranked compounds remain chemically anchored in the thiazolidinone design space without being trivial replicas of the training molecules.

## Conclusions

As development of in silico approaches continue to expand across cheminformatics and drug design, the present results have not reached the promised level yet, indicating the need for paradigm shift in building reliable predictive and generative models. In this work, we address this limitation by enriching classical, well-performing ML frameworks with an AC-aware fragment-based strategy, implemented as the CAFE and CAFE LATE algorithms. Unlike previously reported AC-aware methods, such as SiamACLoss and ACTriplet, which increase AC sensitivity during model training, or MoleculeACE, which formalizes cliff-centered benchmarking and evaluation, our framework aims the same problem from the perspective of drug design logic.

The proposed approach explicitly detects AC pairs, mines fragments hidden within steep SAR regions, removes fragments falsely identified as activity-driving, and recovers overlooked fragments that may promote activity, feeding this corrected fragment knowledge back into de novo molecular generation. Using this AC-aware design logic, we generated and evaluated thiazolidinone derivatives in a focused chemical space relevant to NSCLC. Among 282 unique molecules docked against multiple molecular targets, 241 protein–ligand complexes achieved predicted binding affinities superior to their native ligands, indicating favorable intermolecular interactions. These candidates were further subjected to molecular dynamics simulations to assess the stability of ligand binding within the active site.

Across docking and molecular dynamics benchmarks, CAFE LATE consistently produced a higher proportion of candidates with stable binding modes than the vanilla QSAR-guided baseline. This effect was particularly evident in structural stability metrics, including pocket RMSD and RMSF, where AC-aware candidates more frequently outperformed native references. More importantly, the AC-aware framework generated the strongest individual hits, characterized by simultaneous improvements across multiple metrics: pocket RMSD, pocket RMSF, ligand RMSD, ligand RMSF, and binding energy. Compounds 239 and 343 emerged as the most promising examples, exhibiting both favorable binding energies (− 7.78 and − 7.70 kcal/mol, respectively, compared to native ligand -4.09 kcal/mol) and stable protein–ligand interactions. Importantly, these values are interpreted here in a comparative, protocol-dependent manner and are meant to indicate relative trends rather than absolute binding affinities of studied molecules.

These results suggest that incorporating AC-aware fragment logic into small-molecule generative frameworks can enhance exploration of unknown areas of the chemical space, reduce systematic design errors caused by misleading SAR generalization, and enrich the region of highest-quality candidates with increased likelihood of experimental success. At the same time, the present study is limited by its fully computational nature, reliance on docking and force-field based MD approximations, and restriction to a single chemical scaffold and a predefined set of protein targets. While all evaluations reported here were conducted following current community best practices for data curation and benchmarking, future work will focus on experimental validation and on extending the proposed framework to additional scaffolds and biological targets to further test its generality and practical utility.

## Supplementary Information

Supplementary Material 1.

Supplementary Material 2.

Supplementary Material 3.

Supplementary Material 4.

Supplementary Material 5.

Supplementary Material 6.

Supplementary Material 7.

Supplementary Material 8.

## Data Availability

*De novo* thiazolidinones generative pipeline and CAFE/CAFE LATE architecture: (https://github.com/TomaszSzostek/AC_aware_modeling.git) Molecular docking: (https://github.com/TomaszSzostek/Molecular_docking.git) Molecular dynamics and final leads selection: (https://github.com/maciejwisniewski-drugdiscovery/MolecularDynamicsPipeline/tree/main) The datasets supporting the conclusions of this article are included within the article (and its additional files).
