# Supplementary material for: De novo design of anticancer 4-thiazolidinone derivatives: a generative framework shaped by activity cliffs
Source: J Cheminform. 2026 May 11;18:88. doi: 10.1186/s13321-026-01216-3 (PMC13335218; doi:10.1186/s13321-026-01216-3)
Supplement: Supplementary file 8 — Supplementary Material 8. [file 13321_2026_1216_MOESM8_ESM.docx]

**Supporting Information to:**

**De Novo Design of Anticancer 4-Thiazolidinone Derivatives: a Generative Framework Shaped by Activity Cliffs**

**Tomasz Szostek^a*^, Maciej Wiśniewski^b^, Davide Ballabio^c^, Viviana Consonni^c^, Dariusz Plewczyński^b,d^, Daniel Szulczyk^a^**

*^a^ Chair and Department of Biochemistry, Medical University of Warsaw, 02-097 Warsaw, Poland*

*^b^ Laboratory of Bioinformatics and Computational Genomics, Faculty of Mathematics and Information Science, Warsaw University of Technology, Koszykowa 75, 00-662, Warsaw, Poland*

*^c^ Milano Chemometrics and QSAR Research Group, Department of Earth and Environmental Sciences, University of Milano Bicocca, Piazza della Scienza 1, 20126, Milano, Italy*

*^d^ Laboratory of Functional and Structural Genomics, Centre of New Technologies, University of Warsaw, Banacha 2c, 02-097, Warsaw, Poland*


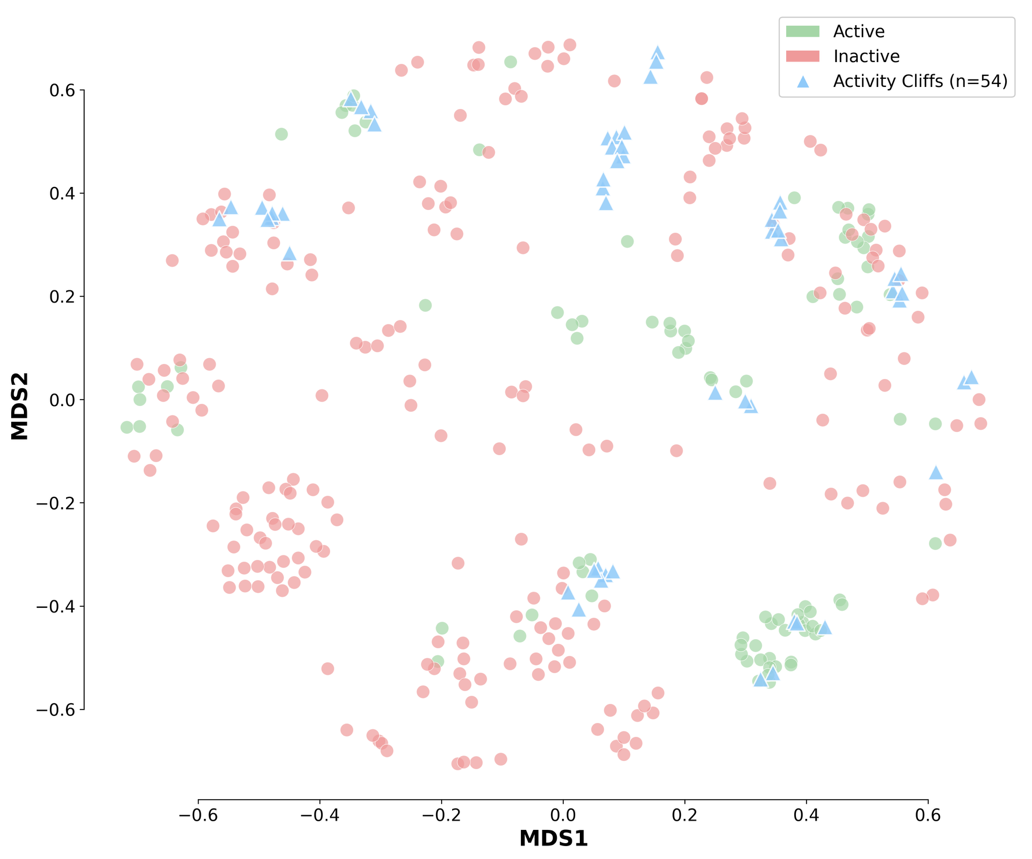


**Figure S.1** Chemical Space projection using Multidimensional Scaling (MDS), computed on ECFP4 Tanimoto Similarity and pIC_50_.

**Table S.1** The summary of mean performance from ten independent data splits of different r-QSAR models

| **Model** | **Performance** | | | |
| --- | --- | --- | --- | --- |
|  | **AUROC** | **AUPRC active** | **MCC** | **Brier** |
| **LogReg** | 0.928  (95% CI, 0.907-0.946) | **0.889**  (95% CI, 0.856-0.917) | 0.746  (95% CI, 0.679-0.808) | 0.112  (95% CI, 0.103-0.122) |
| **CatBoost** | 0.918  (95% CI, 0.894-0.938) | 0.874  (95% CI, 0.840-0.906) | 0.761  (95% CI, 0.714-0.807) | 0.093  (95% CI, 0.078-0.108) |
| **KNN** | 0.915 (95% CI,  0.881-0.941) | 0.861  (95% CI, 0.820-0.892) | 0.735  (95% CI, 0.688-0.780) | 0.100  (95% CI, 0.091-0.112) |
| **GB** | 0.908  (95% CI, 0.881-0.930) | 0.858  (95% CI, 0.822-0.892) | 0.727  (95% CI, 0.676-0.784) | 0.101  (95% CI, 0.087-0.114) |
| **ExtraTrees** | 0.874  (95% CI, 0.829-0.911) | 0.828  (95% CI, 0.773-0.877) | 0.705  (95% CI, 0.621-0.782) | 0.170  (95% CI, 0.164-0.177) |
| **BRF** | 0.861  (95% CI, 0.812-0.902) | 0.816  (95% CI, 0.756-0.868) | 0.686  (95% CI, 0.613-0.760) | 0.181  (95% CI, 0.176-0.187) |
| **SVC** | 0.913  (95% CI, 0.897-0.931) | 0.811  (95% CI, 0.760-0.861) | 0.757  (95% CI, 0.710-0.802) | 0.098  (95% CI, 0.088-0.107) |
| **XGB** | 0.843  (95% CI, 0.800-0.880)" | 0.762  (95% CI, 0.706-0.814) | 0.642  (95% CI, 0.563-0.720) | 0.131  (95% CI, 0.117-0.147) |
| **RF** | 0.842  (95% CI, 0.799-0.881) | 0.751  (95% CI, 0.693-0.811) | 0.568  (95% CI, 0.498-0.644) | 0.192  (95% CI, 0.187-0.197) |

**Table S.2** The final fragments dataset obtained from r-QSAR. Fragments highlighted in blue were added, in orange removed by AC aware CAFE algorithm.

|  | **Fragment SMILES** | **Fragment** |
| --- | --- | --- |
| **1** | [*]c1nc2cc([*])ccc2[nH]1 | 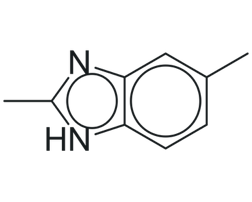 |
| **2** | [*]c1ccc(C#N)cc1 | 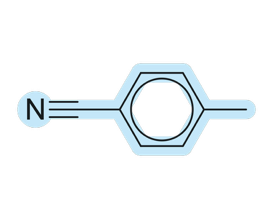 |
| **3** | [*]N1CCCC1 | 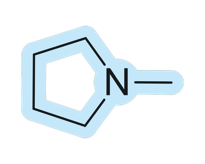 |
| **4** | [*]OC | 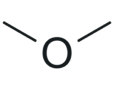 |
| **5** | [*]c1cc2nccc([*])c2cc1[*] | 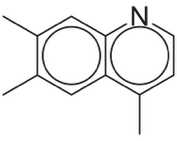 |
| **6** | [*]c1cc([*])c([*])cc1[*] | 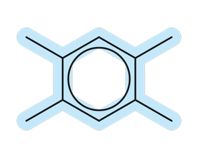 |
| **7** | [*]C(F)(F)F | 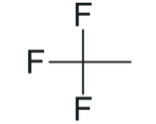 |
| **8** | [*]c1ccc(Br)cc1 | 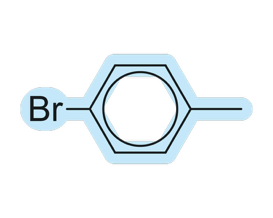 |
| **9** | [*]n1c([*])nc2ccccc21 | 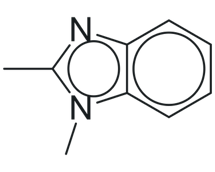 |
| **10** | [*]c1n[nH]c2ccc([*])cc12 | 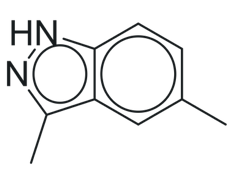 |
| **11** | [*]CC[*] | 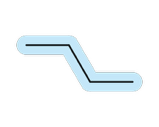 |
| **12** | [*]C1SC(=O)NC1=O | 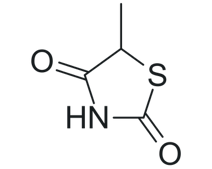 |
| **13** | [*]c1ccc([*])cc1 | 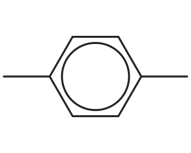 |
| **14** | [*]N1CCCS1(=O)=O | 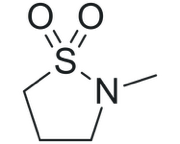 |
| **15** | [*]N1/C(=N/c2ccc(Cl)c([*])c2)SCC1([*])O | 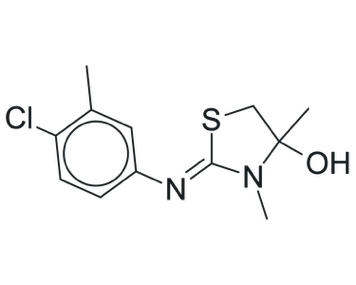 |
| **16** | [*]C(=O)C[*] | 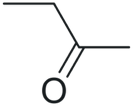 |
| **17** | [*]C[*] | 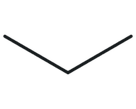 |
| **18** | [*]C(=O)NN1C(=O)CSC1[*] | 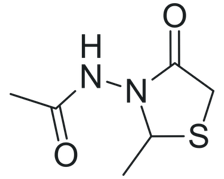 |
| **19** | [*]O[*] | 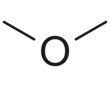 |
| **20** | [*]C([*])=O | 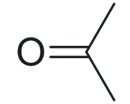 |
| **21** | [*]c1ccc([*])c(F)c1 | 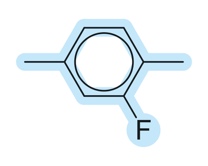 |
| **22** | [*]C1SC(=O)N([*])C1=O | 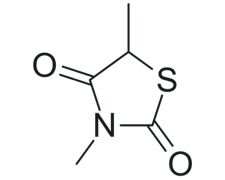 |
| **23** | [*]c1ccc(O)cc1 | 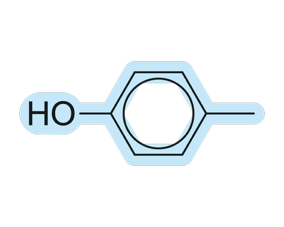 |
| **24** | [*]c1ccccc1F | 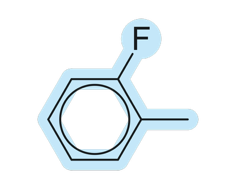 |
| **25** | [*]N1CCN(C)CC1 | 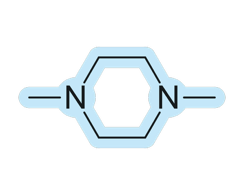 |
| **26** | [*]N1CCOCC1 | 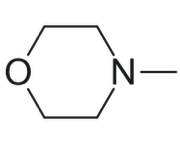 |
| **27** | [*]c1c(F)cccc1F | 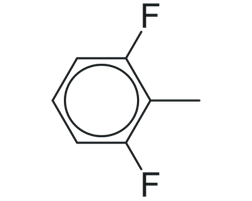 |
| **28** | [*]C1SC(=S)N([*])C1=O | 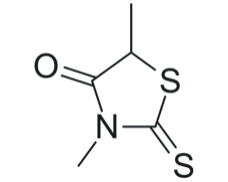 |
| **29** | [*]N[*] | 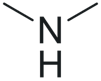 |
| **30** | [H][*] | 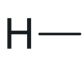 |
| **31** | [*]CCC[*] | 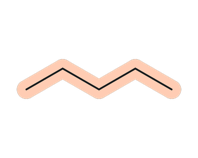 |
| **32** | [*]c1ccc(C)cc1 | 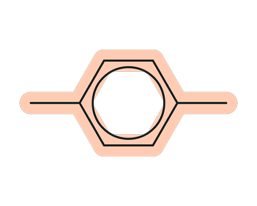 |

**Table S.3** The summary of mean performance from ten independent data splits of different classification QSAR models.

| **Model** | **Performance** | | | |
| --- | --- | --- | --- | --- |
|  | **RMSE_cliff_** | **AUPRC_active** | **AUPRC_cliff_** | **F1_cliff_** |
|  | **ECFP1024** | | | |
| **LogReg** | 0.409  (95% CI, 0.267-0.534) | 0.860  (95% CI, 0.814-0.899) | 0.799  (95% CI, 0.664-0.906) | 0.641  (95% CI, 0.460-0.802) |
| **KNN** | 0.472  (95% CI, 0.338-0.571) | 0.812  (95% CI, 0.765-0.852) | 0.710  (95% CI, 0.557-0.843) | 0.534  (95% CI, 0.294-0.760) |
| **SVC** | 0.437  (95% CI, 0.352-0.515) | 0.799  (95% CI, 0.743-0.849) | 0.789  (95% CI, 0.644-0.904) | 0.684  (95% CI, 0.517-0.825) |
| **RF** | 0.444  (95% CI, 0.348-0.507) | 0.822  (95% CI, 0.784-0.857) | 0.766  (95% CI, 0.643-0.873) | 0.517  (95% CI, 0.287-0.724) |
| **ExtraTrees** | 0.435  (95% CI, 0.333-0.501) | 0.837  (95% CI, 0.790-0.881) | 0.797  (95% CI, 0.663-0.911) | 0.654  (95% CI, 0.463-0.822) |
| **GB** | 0.416  (95% CI, 0.284-0.532) | 0.856  (95% CI, 0.809-0.898) | 0.805  (95% CI, 0.673-0.912) | 0.678  (95% CI, 0.512-0.823) |
| **XGB** | 0.420  (95% CI, 0.299-0.524) | 0.860  (95% CI, 0.815-0.900) | 0.784  (95% CI, 0.650-0.889) | 0.674  (95% CI, 0.491-0.823) |
| **CatBoost** | 0.438  (95% CI, 0.281-0.571) | 0.812  (95% CI, 0.757-0.860) | 0.750  (95% CI, 0.617-0.865) | 0.655  (95% CI, 0.486-0.810) |
| **BRF** | 0.440  (95% CI, 0.343-0.503) | 0.836  (95% CI, 0.794-0.874) | 0.808  (95% CI, 0.674-0.916) | 0.697  (95% CI, 0.515-0.845) |
|  | **ECFP2048** | | | |
| **LogReg** | 0.409  (95% CI, 0.2680.536) | 0.864  (95% CI, 0.819-0.901) | 0.806  (95% CI, 0.671-0.914) | 0.666  (95% CI, 0.517-0.810) |
| **KNN** | 0.477  (95% CI, 0.3450.573) | 0.810  (95% CI, 0.765-0.849) | 0.709  (95% CI, 0.554-0.845) | 0.597  (95% CI, 0.374-0.785) |
| **SVC** | 0.437  (95% CI, 0.3510.515) | 0.792  (95% CI, 0.734-0.841) | 0.777  (95% CI, 0.634-0.897) | 0.700  (95% CI, 0.570-0.824) |
| **RF** | 0.444  (95% CI, 0.349-0.506) | 0.822  (95% CI, 0.782-0.858) | 0.793  (95% CI, 0.650-0.908) | 0.618  (95% CI, 0.413-0.787) |
| **ExtraTrees** | 0.440  (95% CI, 0.344-0.503) | 0.820  (95% CI, 0.773-0.863) | 0.793  (95% CI, 0.655-0.905) | 0.631 (95% CI, 0.442-0.796) |
| **GB** | 0.415  (95% CI, 0.281-0.531) | 0.852  (95% CI, 0.802-0.896) | 0.772  (95% CI, 0.622-0.900) | 0.690  (95% CI, 0.534-0.832) |
| **XGB** | 0.417  (95% CI, 0.294-0.524) | 0.845  (95% CI, 0.797-0.894) | 0.774  (95% CI, 0.633-0.900) | 0.699  (95% CI, 0.546-0.842) |
| **CatBoost** | 0.430  (95% CI, 0.275-0.563) | 0.805  (95% CI, 0.737-0.862) | 0.735  (95% CI, 0.590-0.861) | 0.685  (95% CI, 0.539-0.822) |
| **BRF** | 0.441  (95% CI, 0.344-0.506) | 0.819  (95% CI, 0.772-0.859) | 0.793  (95% CI, 0.652-0.906) | 0.524  (95% CI, 0.317-0.724) |
|  | **MACCS** | | | |
| **LogReg** | 0.411  (95% CI, 0.272-0.530) | 0.812  (95% CI, 0.763-0.854) | 0.790  (95% CI, 0.647-0.895) | 0.668  (95% CI, 0.509-0.816) |
| **KNN** | 0.483  (95% CI, 0.364-0.571) | 0.804  (95% CI, 0.767-0.840) | 0.705  (95% CI, 0.559-0.843) | 0.529  (95% CI, 0.292-0.753) |
| **SVC** | 0.450  (95% CI, 0.356-0.529) | 0.827  (95% CI, 0.774-0.867) | 0.735  (95% CI, 0.611-0.845) | 0.653  (95% CI, 0.508-0.792) |
| **RF** | 0.430  (95% CI, 0.330-0.495) | 0.826  (95% CI, 0.782-0.862) | 0.752  (95% CI, 0.601-0.880) | 0.560  (95% CI, 0.326-0.774) |
| **ExtraTrees** | 0.417  (95% CI, 0.315-0.491) | 0.833  (95% CI, 0.791-0.872) | 0.787  (95% CI, 0.631-0.910) | 0.625  (95% CI, 0.423-0.787) |
| **GB** | 0.421  (95% CI, 0.282-0.540) | 0.867  (95% CI, 0.823-0.906) | 0.790  (95% CI, 0.646-0.903) | 0.687  (95% CI, 0.516-0.829) |
| **XGB** | 0.425  (95% CI, 0.299-0.532) | 0.855  (95% CI, 0.813-0.894) | 0.739  (95% CI, 0.601-0.855) | 0.560  (95% CI, 0.362-0.731) |
| **CatBoost** | 0.436  (95% CI, 0.286-0.565) | 0.839  (95% CI, 0.797-0.874) | 0.752  (95% CI, 0.598-0.877) | 0.693  (95% CI, 0.538-0.827) |
| **BRF** | **0.421**  **(95% CI, 0.324-0.487**) | 0.831  (95% CI, 0.792-0.866) | 0.776  (95% CI, 0.625-0.900) | 0.604  (95% CI, 0.402-0.779) |
|  | **Descriptors** | | | |
| **LogReg** | 0.403  (95% CI, 0.248-0.534) | 0.863  (95% CI, 0.824-0.893) | 0.803  (95% CI, 0.695-0.897) | 0.677  (95% CI, 0.510-0.822) |
| **KNN** | 0.410  (95% CI, 0.283-0.510) | 0.813  (95% CI, 0.766-0.855) | 0.749  (95% CI, 0.599-0.874) | 0.714  (95% CI, 0.573-0.843) |
| **SVC** | 0.432  (95% CI, 0.334-0.519) | 0.851  (95% CI, 0.810-0.887) | 0.727  (95% CI, 0.589-0.850) | 0.657  (95% CI, 0.528-0.771) |
| **RF** | 0.428  (95% CI, 0.305-0.525) | 0.831  (95% CI, 0.778-0.876) | 0.733  (95% CI, 0.592-0.859) | 0.680  (95% CI, 0.531-0.816) |
| **ExtraTrees** | 0.429  (95% CI, 0.331-0.499) | 0.833  (95% CI, 0.783-0.872) | 0.761  (95% CI, 0.624-0.872) | 0.667  (95% CI, 0.511-0.816) |
| **GB** | 0.423  (95% CI, 0.267-0.555) | 0.855  (95% CI, 0.806-0.896) | 0.763  (95% CI, 0.627-0.875) | 0.638  (95% CI, 0.490-0.786) |
| **XGB** | 0.426  (95% CI, 0.291-0.542) | 0.839  (95% CI, 0.790-0.882) | 0.743  (95% CI, 0.622-0.854) | 0.654  (95% CI, 0.524-0.789) |
| **CatBoost** | 0.424  (95% CI, 0.260-0.562) | 0.852  (95% CI, 0.807-0.897) | 0.782  (95% CI, 0.654-0.895) | 0.667  (95% CI, 0.536-0.806) |
| **BRF** | 0.424  (95% CI, 0.298-0.524) | 0.832  (95% CI, 0.784-0.874) | 0.734  (95% CI, 0.605-0.855) | 0.674  (95% CI, 0.536-0.812) |

**Table S.4** Summarized min|max energies from twelve poses of redocked native ligands with best RMSD values.

| **PDB Crystal Structure** | **Ligand**  **name** | **Biding Energy (min\|max) [kcal/mol]** | **RMSD (Å)** |
| --- | --- | --- | --- |
| 1M17 | AQ4 | -7.1 \| -6.3 | 1.551 |
| 4I23 | 1C9 | -8.1 \| -7.2 | 0.234 |
| 8GUA | 1LT | -9.1 \| -7.8 | 1.312 |
| 4EKL | 0RF | -9.7 \| -7.8 | 0.938 |
| 5OQ4 | A3W | -9.1 \| -8.0 | 0.905 |
| 4LMN | EUI | -11.8 \| -9.6 | 0.738 |
| 5HD4 | 38Z | -14.2 \| -11.2 | 0.135 |
| 6SCM | L7H | -9.8 \| -7.3 | 0.773 |
| 5EHR | 5OD | -10.7 \| -7.7 | 0.101 |
| 4L7B | 1VV | -10.8 \| -8.2 | 0.233 |
| 5L2S | 6ZV | -10.2 \| -9.1 | 0.595 |
| 5L2I | LQQ | -10.2 \| -7.9 | 0.520 |
| 5L2T | 6ZZ | -10.4 \| -7.7 | 0.121 |


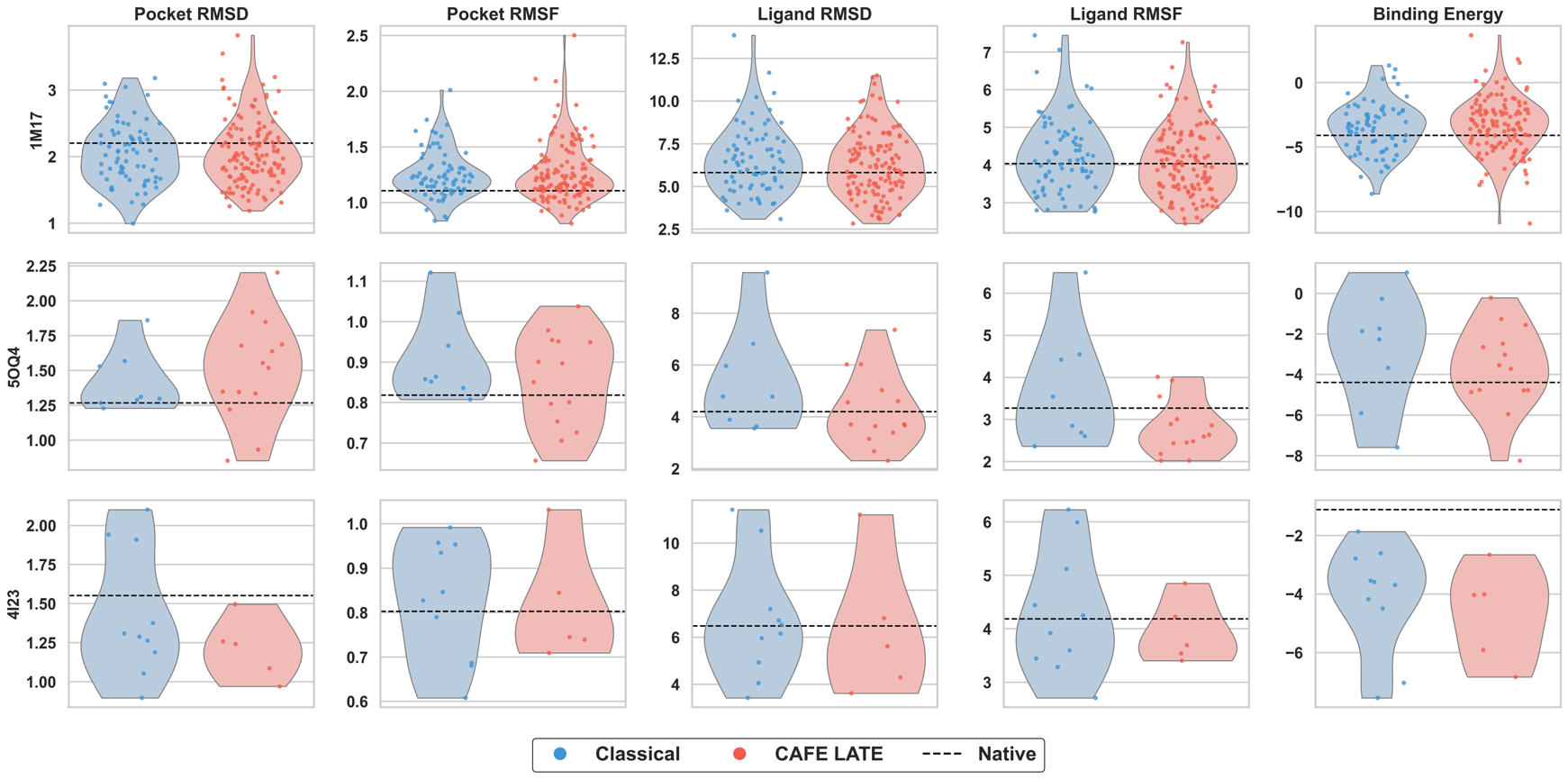
**Figure S2.** Distribution of metrics across proteins and methods.


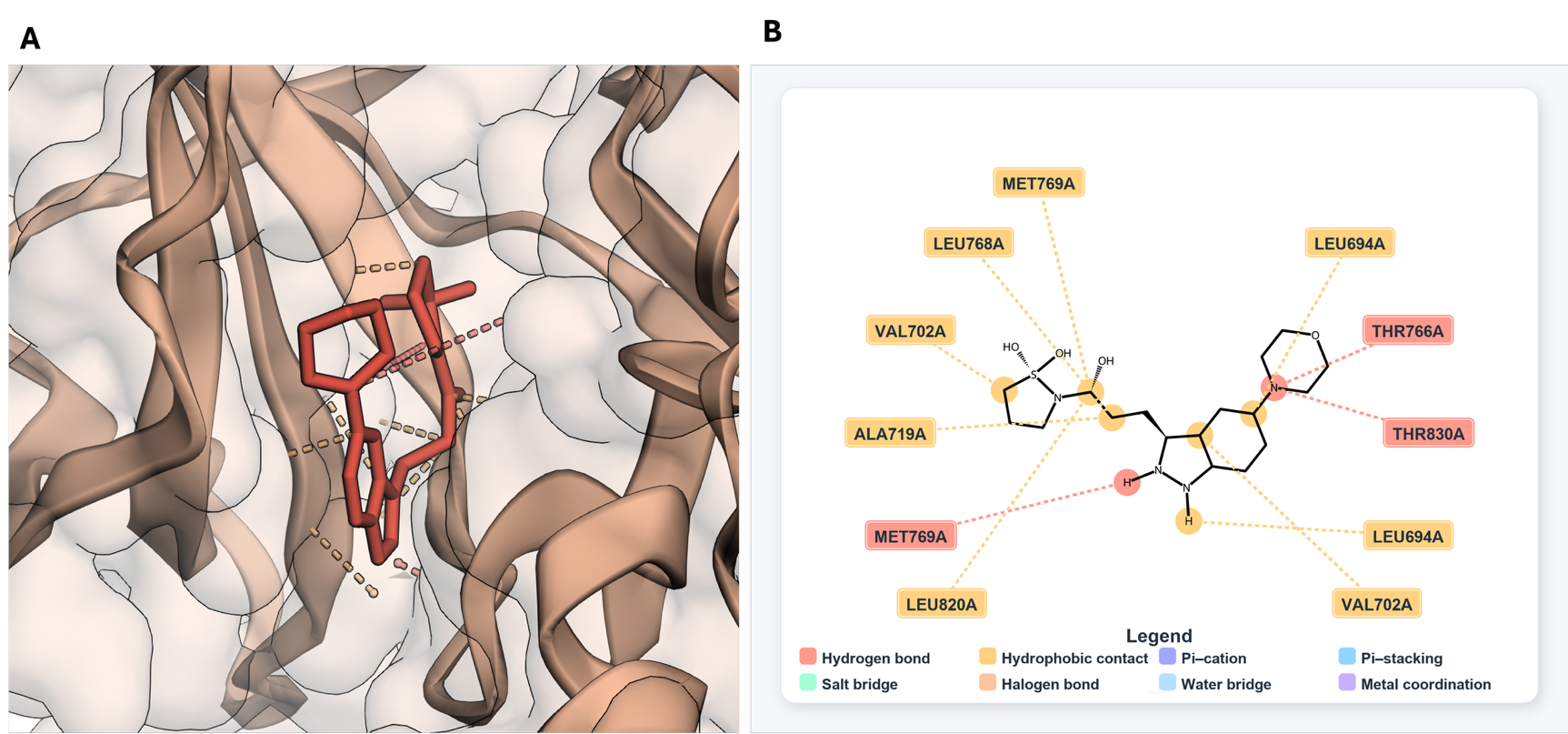


**Figure S3.** Protein-ligand **(A)** 2D- and **(B)** 3D- interactions from molecular docking analysis of compound **239** with 1M17.
